# Oncolytic Viruses and Immunotherapy for the Treatment of Uveal Melanoma and Retinoblastoma: The Current Landscape and Novel Advances

**DOI:** 10.3390/biomedicines13010108

**Published:** 2025-01-06

**Authors:** Merve Kulbay, Nicolas Tuli, Massimo Mazza, Armaan Jaffer, Sarinee Juntipwong, Emily Marcotte, Stuti Misty Tanya, Anne Xuan-Lan Nguyen, Miguel N. Burnier, Hakan Demirci

**Affiliations:** 1 Department of Ophthalmology & Visual Sciences, McGill University, Montreal, QC H4A 3J1, Canada; merve.kulbay@mail.mcgill.ca (M.K.);; 2 Faculty of Medicine and Health Sciences, McGill University, Montreal, QC H4A 3J1, Canada; 3 Faculty of Health Sciences, Queen’s University, Kingston, ON K7L 2V5, Canada; 4 Research Excellence Cluster in Vision, University of British Columbia, Vancouver, BC V5Z 3N9, Canada; 5 Kellogg Eye Center, Department of Ophthalmology and Visual Science, University of Michigan, Ann Arbor, MI 48105, USA; 6 McGill University Ocular Pathology and Translational Research Laboratory, McGill University, Montreal, QC H4A 3J1, Canada; emily.marcotte@mail.mcgill.ca; 7 Cancer Research Program, Research Institute of the McGill University Health Centre, Montreal, QC H4A 3J1, Canada; 8 Department of Ophthalmology & Vision Sciences, University of Toronto, Toronto, ON M4N 3M5, Canada

**Keywords:** uveal melanoma, retinoblastoma, advances in oncolytic viruses and immunotherapy, intraocular tumors

## Abstract

Intraocular malignant tumors are rare; however, they can cause serious life-threatening complications. Uveal melanoma (UM) and retinoblastoma (RB) are the most common intraocular tumors in adults and children, respectively, and come with a great disease burden. For many years, several different treatment modalities for UM and RB have been proposed, with chemotherapy for RB cases and plaque radiation therapy for localized UM as first-line treatment options. Extraocular extension, recurrence, and metastasis constitute the major challenges of conventional treatments. To overcome these obstacles, immunotherapy, which encompasses different treatment options such as oncolytic viruses, antibody-mediated immune modulations, and targeted immunotherapy, has shown great potential as a novel therapeutic tool for cancer therapy. These anti-cancer treatment options provide numerous advantages such as selective cancer cell death and the promotion of an anti-tumor immune response, and they prove useful in preventing vision impairment due to macular and/or optic disc involvement. Numerous factors such as the vector choice, route of administration, dosing, and patient characteristics must be considered when engineering an oncolytic virus or other forms of immunotherapy vectors. This manuscript provides an in-depth review of the molecular design of oncolytic viruses (e.g., virus capsid proteins and encapsulation technologies, vectors for delivery, cell targeting) and immunotherapy. The most recent advances in preclinical- and clinical-phase studies are further summarized. The recent developments in virus-like drug conjugates (i.e., AU011), oncolytic viruses for metastatic UM, and targeted immunotherapies have shown great results in clinical trials for the future clinical application of these novel technologies in the treatment algorithm of certain intraocular tumors.

## 1. Introduction

Uveal melanoma (UM) and retinoblastoma (RB) are the most common intraocular tumors in adults and children, respectively, [1]. Together, they are responsible for 15,000 new cases per year around the world [1]. Oncolytic viruses (OVs) and immunotherapy—including targeted immunotherapy—have recently gained interest as novel therapeutic agents for the management of UM and RB. Unlike conventional treatment modalities, such as radiotherapy and chemotherapy, OVs are thought to have improved safety profiles in vision preservation. In this review, we discuss the engineering process of OVs and the different types of immunotherapies, as well as summarize the novel advances in the related fields.

## 2. Overview of Uveal Melanoma and Retinoblastoma’s Pathogenesis

Tumorigenesis is characterized by the spontaneous induction of genetic mutations which drive the uncontrolled proliferation of abnormal cells. However, tumor development involves a far more intricate interplay of genetic, molecular, and environmental factors. Studies have shown the importance of immune dysregulations and abnormalities within the apoptotic and inflammatory pathways, as well as the tumor microenvironment (TME), in UM [2,3,4,5] and RB [6,7,8,9,10] tumorigenesis. With the development of personalized medicine, novel treatment options aim to target specific cellular pathways that promote tumor development. In this section, we will present an overview of altered cellular pathways in UM and RB that are subject to targeting with the novel treatment modalities. We recommend referring to the cited references for further details on UM and RB tumorigenesis.

### 2.1. Uveal Melanoma Tumorigenesis Overview

**Figure 1:**
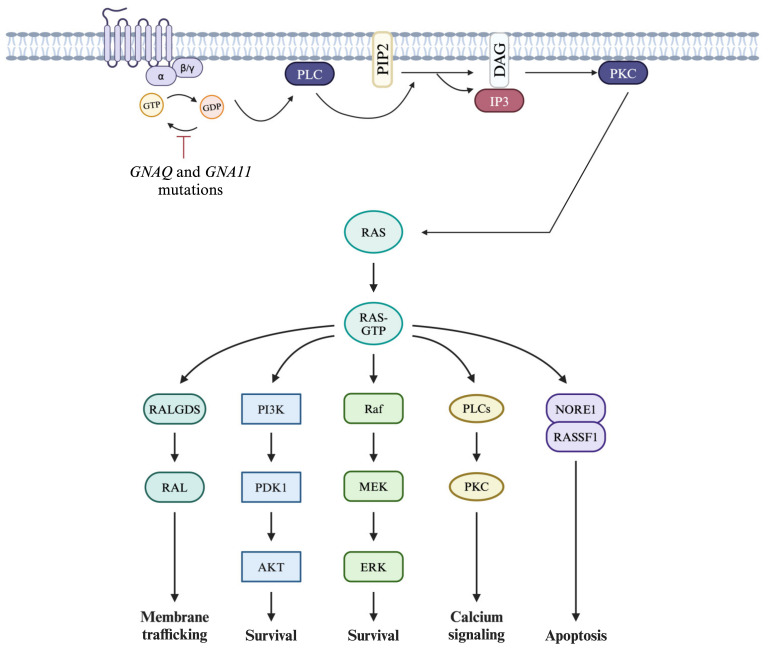
Downstream cellular signaling dysregulations involved in the pathogenesis of uveal melanoma following *GNAQ* and *GNA11* mutations. *GNAQ* and *GNA11* mutations lead to the uncontrolled activation of G protein signaling pathways, ultimately inducing cancer cell survival, proliferation, and the inhibition of apoptosis. Created in BioRender. Kulbay, M. (2024) https://BioRender.com/d29b243.

*GNAQ* and *GNA11* mutations are present in up to 90% of UM cases [11]. These genes are involved in the regulation of the Gα subunits of G proteins. Mutations in *GNAQ*/*GNA11* have been shown to lead to the uncontrolled activation of G proteins, leading to the excessive downstream signaling of molecular partners shown in Figure 1. Silva-Rodríguez et al. have recently thoroughly reviewed *GNAQ*/*GNA11*-mediated UM oncogenesis, and we suggest referring to this review for detailed explanations [11]. Briefly, the RAS/RAF/MEK/ERK/MAPK signaling pathway is mainly involved in UM tumorigenesis. Downstream signaling pathways are involved in uncontrolled cell growth, cell proliferation, cell survival, angiogenesis, and apoptosis resistance [11].

### 2.2. Retinoblastoma Tumorigenesis Overview

**Figure 2:**
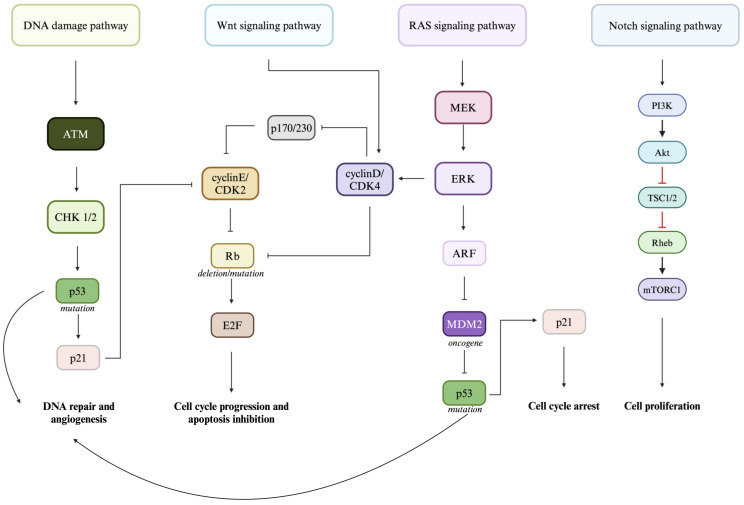
Schematic illustration of pathways altered in retinoblastoma pathogenesis. Four main pathways are involved in retinoblastoma (Rb) pathogenesis: DNA damage pathways and Wnt signaling, RAS signaling, and Notch signaling pathways. Mutations in gene expression lead to disruptions in downstream signalization. Created in BioRender. Kulbay, M. (2024) https://BioRender.com/x11l167.

The pathogenesis of RB is complex and driven by mutations in the *RB1* gene, a tumor suppressor gene, that can either be sporadic (somatic mutations) or heritable (germline mutations) [12]. Genetic and post-translational modifications driving *RB1*-mediated tumorigenesis have recently been thoroughly reviewed by Zhou et al. [13]. These genetic abnormalities are subsequently involved in the dysregulation of four major signaling pathways constituting the backbone of RB pathogenesis, DNA damage signaling, Wnt signaling, Ras signaling, and Notch signaling (Figure 2), as explained by Byroju et al. [14]. However, it is important to mention that RB tumorigenesis is highly complex: numerous additional oncogenic mutations have been shown to be involved in RB tumorigenesis [12].

## 3. Current Therapeutic Approaches

### 3.1. Conventional Treatment Modalities for Uveal Melanoma

The current therapeutic landscape for UM includes several options such as plaque radiotherapy or proton beam radiotherapy (PBT), enucleation, and transpupillary thermotherapy (TTT) or local resection in selected cases (see Table 1) [15]. The treatment strategy centers around the size and location of the tumor and the prospect of salvaging the vision [16]. Radiotherapy, including plaque brachytherapy and PBT, is the most utilized therapeutic approach in the management of UM [17,18]. Enucleation is the most common surgery for patients with large tumors [17,19]. Local resection is usually for iris and ciliary body melanomas rather than choroidal melanomas, and laser techniques, such as TTT and photodynamic therapy (PDT), play less prominent roles in the treatment of UM.

### 3.2. Conventional Treatment Modalities for Retinoblastoma

With several treatment modalities available, RB management is complex [32,33]. Management is often comprehensive, integrating diverse approaches with consideration given to the tumor laterality, size of the tumor, vitreous or subretinal seeding, extraocular involvement, vision potential, patient age and health, and family preferences [32,34,35,36]. While therapeutic protocols may differ across centers worldwide, the core principles include preserving life by preventing metastasis, salvaging the eye, and optimizing visual acuity. However, despite significant advancements in treatment over the past decades, the chance of tumor recurrence and metastasis persists [37]. Chemotherapy is typically the backbone of a multi-component treatment strategy [6,35]. Although chemotherapeutic approaches can be employed independently, they are typically used in combination with other modalities, such as focal laser or radiation therapy [35]. Treatment modalities, their indications, and disadvantages are summarized in Table 2.

### 3.3. Emerging Therapeutic Approaches

Although modern interventions for UM and RB have significantly improved the patient prognosis, the risk of tumor recurrence remains a notable concern [15,37]. Additionally, conventional treatment modalities can lead to long-lasting side effects, including ocular toxicity and systemic complications, while certain RB tumors have developed resistance to chemotherapeutic approaches [6,15,36,66]. The emergence of OVs and immunotherapy offers the potential to change the treatment landscape for these intraocular tumors, given their targeted mechanisms, lack of toxic complications, and potential for a synergistic impact [67,68,69,70,71,72,73]. OVs are genetically engineered such that they selectively infect and lyse malignant cells without harming normal tissues. Immunotherapy, which includes modalities such as immune checkpoint inhibitors (ICIs) and adoptive T cell therapy (ATT), facilitates a strengthened immune response, allowing immune cells to target and destroy malignancies more effectively [73]. The concurrent application of these two treatments not only fosters a synergistic enhancement of the immune system’s capacity but also demonstrates significant potential for integration with current treatments like chemotherapy [73,74,75].

## 4. Oncolytic Viruses

OVs offer a diverse and highly manipulatable strategy to target cancer cells. As with several viral species, their capsid proteins and replication mechanisms can be optimized to enhance tumor recognition. Furthermore, OVs can be genetically enhanced to increase their anti-tumorigenic effects, which include oncolysis, boosting immune responses, and manipulating the TME. Currently, numerous virus-based oncolytic and drug delivery systems are under investigation, such as AU-011, a virus-like drug conjugate (VDC) that has gained interest over the past few years. Oncolytic viruses have also shown great efficiency in the treatment of metastatic UM, which is discussed in Section 6.

### 4.1. Mechanism of Action

**Figure 3:**
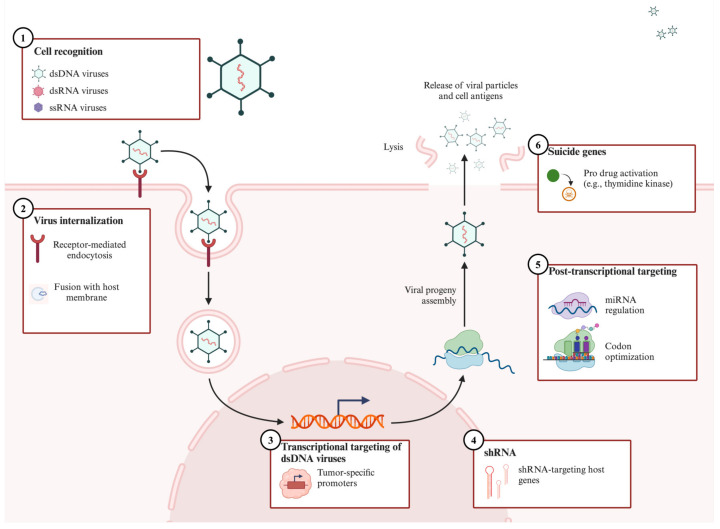
Schematic illustration of the mechanism of action of oncolytic viruses. Oncolytic viruses (OVs), which can be dsDNA-, dsRNA-, or ssRNA-based, interact with cancerous cells through surface proteins or fibers that recognize the extracellular receptors. The OV is internalized through receptor-mediated endocytosis or through cell membrane fusion. Once internalized, dsDNA OVs translocate to the nucleus where their genome is integrated within the host DNA to induce the transcription of viral proteins. RNA-based OVs (not represented in this illustration) undergo replication within the cytoplasm. Upon viral progeny assembly, viral particles and cell antigens are expulsed into the tumor microenvironment, leading to cell oncolysis and the continuation of the infectious cycle. Created in BioRender. Kulbay, M. (2024) https://BioRender.com/w63e114.

The primary pathway for tumor elimination is known as oncolysis (Figure 3). OVs infect tumor cells through the interaction of viral coat proteins with target cell receptors [76]. The OVs’ surface proteins or fibers recognize extracellular receptors expressed on both cancerous and healthy host cells [77]. The OVs can be internalized through multiple mechanisms, which further leads to the release of their genome into the cytoplasm in RNA-based viruses or translocation to the nucleus to induce transcriptional changes [70,78]. The aim of the intracellular virus is to produce a plethora of viral progeny that will lyse the cell, a process called oncolysis. OVs are pathogenic and lead to innate and adaptative immune responses to viral DNA, RNA, and proteins [79]. These immune responses lead to the clearance of the virus, which can impact the efficiency of the treatment. Furthermore, oncolysis exposes both viral and tumor-associated antigens, which leads to immune responses directed against tumor cells. Both the oncolytic and immune-triggering properties of an OV can be modified or enhanced to improve OVs’ anti-tumorigenic properties.

### 4.2. Engineering of Oncolytic Viruses

Engineering an oncolytic virus (OV) for therapeutic applications in intraocular tumors involves a multi-step process. This begins with selecting an appropriate virus subtype, followed by making biochemical alterations and optimizing the virus’s properties. Another critical consideration is determining the most effective route of administration. Throughout this process, pharmaceutical development must balance cost-effectiveness, clinical efficiency, and the minimization of side effects.

#### 4.2.1. Species and Types of Viruses

All OVs share common traits but vary in their genome and structures, predominantly in terms of viral capsid and envelope proteins, resulting in different types of OVs with varying effectiveness against specific tumors. OVs can be divided into three distinct categories based on their genetic material: (i) single-stranded (ss) RNA, (ii) double-stranded (ds) RNA, or (iii) dsDNA (Table 3).

dsDNA viruses represent an important class of OVs. Both the Herpes simplex virus (HSV) and Adenovirus (ADV) have been extensively studied for their potential in treating ocular tumors. HSVs exist as two specific serotypes (HSV-1 and HSV-2), while ADV has 57 different serotypes [70,77,80]. A key characteristic of both OVs is their large genomes, allowing for the insertion of transgenes to alter their tumor specificity or tumor elimination capacity [70]. The HSV, an enveloped virus, utilizes four glycoproteins (i.e., gB, gD, gH, and gL) expressed on its viral envelopes to bind specific cellular receptors, facilitating fusion with the cell membrane [81]. Conversely, ADVs are non-enveloped viruses and thus rely on fiber knobs on their capsid to recognize specific receptors triggering endocytosis [70,82]. Once internalized, both OVs’ genomes translocate to the nucleus to integrate with the host’s genome, further producing viral progeny that will ultimately lead to oncolysis.

**Table 3:** Summary of most studied oncolytic viruses for the treatment of intraocular tumors.

Virus Examples	Genome Size	Advantages	Disadvantages	References
Double-strand DNA viruses				
Herpes simplex virus	152 kb	More amenable to genetic manipulation	Neutralizing antibodies in individuals can limit its efficiency	[83,84]
Adenovirus	26 to 45 kb		High tissue tropism	[79,85]
Vaccinia virus	190 kb	Fast and efficient transfection of tumor cells More amenable to genetic manipulation	High pathogenicity to healthy cells	[70,79]
Double-strand RNA viruses				
Reoviruses	16–27 kbp	Unique ability to target junctional adhesion molecule (JAM)-A Easier delivery to target cells	No evidence in intraocular tumors	[86,87]
Single-strand RNA viruses				
Seneca Valley Virus	7.3 kb	Reduced risk of insertional mutagenesis Easier delivery to target cells	Limited efficiency clinically	[70,88,89]
Vesicular stomatitis Indiana virus	11 kb	Highly cytopathic Efficient in tumors with defective interferon signaling pathway Easier delivery to target cells	Neurotoxicity Rapid clearance by the immune system	[90,91,92]

#### 4.2.2. Modulating Tumor Recognition

While OVs possess inherent anti-tumorigenic properties, their therapeutic potential can be significantly optimized through modifications that improve tumor cell recognition and optimize viral replication. This can be achieved by enhancing the binding of OVs to tumor-specific receptors, thereby improving their selectivity and minimizing off-target effects.

**Figure 4:**
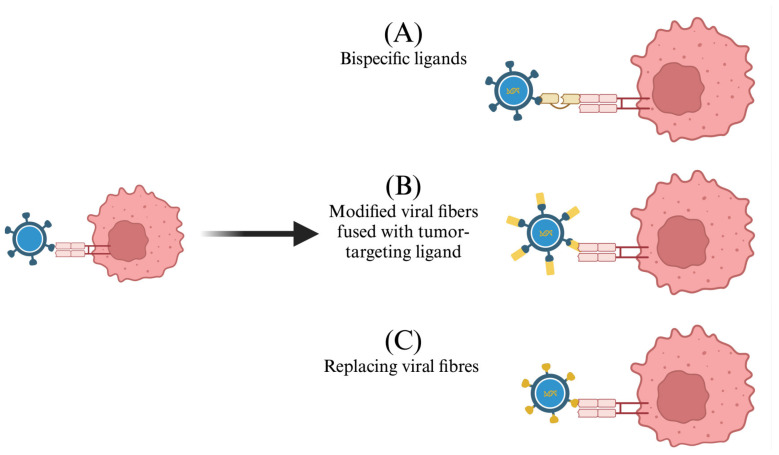
Modifying oncolytic viruses to enhance tumor recognition. (**A**) Oncolytic viruses (OVs) are modified to express bispecific ligands, such as single-chain antibodies, that facilitate the interaction between viral capsid fiber/envelope proteins and their target receptor. (**B**) OVs are modified to have their capsid fibers/envelope fused to a tumor-targeting ligand which enhances tumor-specific protein recognition. (**C**) OVs are modified to have their viral capsid protein/fibers replaced with another virus’s protein to enhance the recognition of specific tumors. Created in BioRender. Tuli, N. (2025) https://BioRender.com/b97r111.

The selective targeting of OVs to cancer cells relies on modifying the interaction between the OVs and tumor-associated cell surface proteins. There are three popular strategies being studied to retarget OVs to specific tumors (Figure 4). The first strategy involves the incorporation of a bispecific ligand that recognizes both the cancer cell surface protein and the ADV fiber knob domains that regulate cell entry [93]. These ligands, often single-chain antibodies, bridge the interaction between virus and tumor cells, resulting in higher rates of internalization, but unfortunately have shown limited success so far [94]. A second approach involves modifying viral capsid or fiber proteins to display tumor-targeting ligands. For example, HSV glycoproteins can be fused to a single-chain antibody specific to tumor-associated proteins that enhance the targeting of OVs to tumor cells [70,95]. A third strategy is to restructure the OV capsid. For example, ADVs can be genetically altered, resulting in the replacement of fiber knobs with those from another serotype or different virus species [70]. Yang et al. demonstrated the potential of this strategy by combining the fiber knobs of two ADV serotypes to enhance the targeting of the OV to specific tumors [96].

The enhancement of OV selectivity can also be achieved through optimizing the OVs’ capabilities to leverage the cellular mechanisms altered in tumor cells. For example, some viruses such as ADVs will rely on certain proteins, such as tumor suppressor protein p53, to replicate in a cell by preventing apoptosis. By inactivating the viral proteins that mediate this interaction, apoptosis can be triggered in healthy cells infected by the OVs [97]. Contrarily, apoptosis will be absent in many cancers with p53 mutations, allowing the virus to selectively replicate and lyse cancerous cells [70,97,98]. Another method involves ablating genes required for the proliferation of OVs. In certain tumors, upregulated proteins can replace the function of the ablated viral proteins, enabling the selective replications of OVs in tumor cells that highly express the required protein [70,99]. Ultimately, these strategies highlight the ongoing efforts to enhance the specificity and efficacy of oncolytic viruses, harnessing their potential to selectively target and destroy cancer cells while minimizing damage to healthy tissues.

#### 4.2.3. Modulating Tumor Elimination

**Figure 5:**
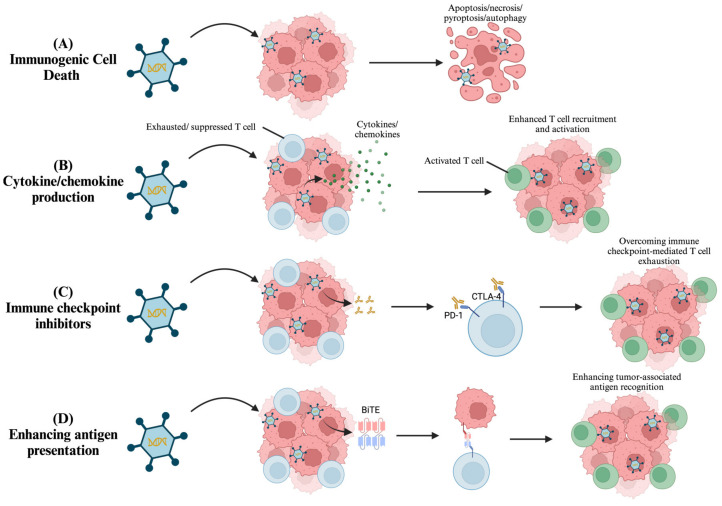
Arming oncolytic viruses to enhance tumor elimination through four mechanisms. (**A**) Oncolytic viruses (OVs) are armed to express pro-immunogenic cell death (ICD) proteins that enhance apoptosis, necrosis, pyroptosis, or autophagy. (**B**) Arming OVs to express chemokines or cytokines, which are immune mediators that recruit T cells (chemokines) or enhance their activation and proliferation (cytokines). (**C**) OVs armed with immune checkpoint inhibitors (ICIs) that interfere with tumor-mediated immunosuppression, leading to enhanced T cell activation and proliferation. (**D**) Arming OVs with proteins such as bispecific T cell engager (BiTE) facilitates the interaction between T cells and tumor-associated antigens, enhancing T cell function. Created in BioRender. Tuli, N. (2025) https://BioRender.com/b97r111.

The therapeutic potential of OVs can also be increased through the modulation of tumor elimination, by acting on cell death pathways, activating immune responses, and by overcoming T cell exhaustion or suppression (Figure 5).

##### Immunogenic Cell Death

The oncolytic activity relies on the OV’s capacity to trigger one of several cell death pathways, including apoptosis, necrosis, pyroptosis, or autophagy, which together contribute to immunogenic cell death (ICD) (Figure 5A). All four pathways can be leveraged when designing oncolytic virotherapies. By genetically engineering OVs to express or upregulate pro-ICD genes, researchers aim to enhance the OV-mediated ICD of tumor cells [100].

##### Activating Immune Responses

**Figure 6:**
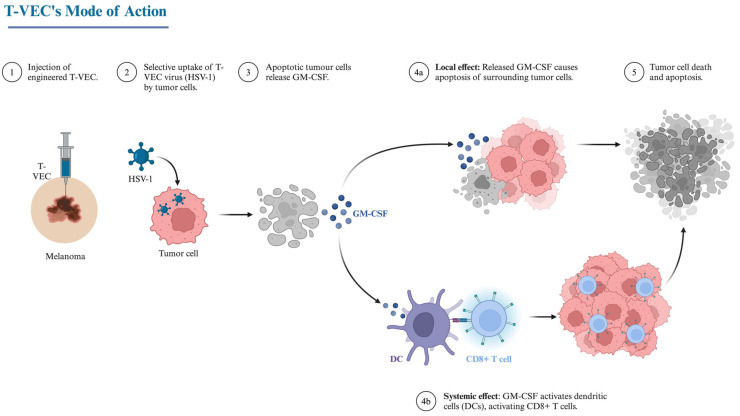
Schematic illustration of the mechanism of action of Talimogene Laherparepvec. Created in BioRender. Kulbay, M. (2024) https://BioRender.com/c38m199.

Several cytokines have been shown to contribute to anti-tumor responses and thus have been investigated as additives to OVs to enhance their therapeutic efficacy (Figure 5B). Granulocyte–macrophage colony-stimulating factor (GM-CSF) is a prime example, in that it promotes the proliferation and activation of dendritic and T cells [101]. The first FDA-approved OV, Talimogene Laherparepvec (T-VEC), encodes GM-CSF, which enhances antigen-presenting cell (APC) recruitment, therefore promoting tumor-specific T cell responses against cutaneous melanomas [102,103] (Figure 6). Other cytokines that have shown promise in enhancing OV-mediated immune responses include ILs [79]. Liu et al. demonstrated that genetically modifying the vaccinia virus to express IL-2 enhanced tumor clearance in mouse models [104]. Furthermore, HSVs modified to encode IL-12, a potent activator of NK and T cells, have shown enhanced anti-tumorigenic effects in several preclinical models [105]. Similar anti-tumor effects have been seen with measles-based OVs that have been modified to express IL-15, which also promotes the activation of NK and CD8 T cells [106]. Lastly, IFNs have also been studied in combination with OVs. Both type I (IFNα and IFNβ) and type II IFNs (IFNγ) are involved in anti-viral immune responses; however, they can also protect against tumor proliferation. IFNα and IFNβ directly act on host hematopoietic cells, resulting in tumor inhibition, whereas IFNγ promotes tumor recognition by upregulating the MHC class I pathway, leading to enhanced T cell activation [107,108]. OVs engineered to express either type I or type II IFNs have been shown to boost immune activity against tumors in preclinical models [108,109]. Overall, these preclinical and clinical successes have demonstrated the promise of engineering OVs to express specific cytokines to enhance their therapeutic efficacy.

Chemokines, similarly to cytokines, are small soluble proteins, but with the distinct function of recruiting immune cells to specific sites. Arming OVs with the ability to produce chemokines, such as chemotactic cytokine CCL5, increases the recruitment of anti-tumor CD8^+^ T cells and NK cells, which express the receptor for CCL5, CCR5 [110]. This chemotactic strategy was shown to improve the anti-tumor effects of OVs [110]. A similar strategy using CXCL11-armed OVs led to increased CD8^+^ T cell recruitment, which also resulted in an increased anti-tumor effect [111].

##### Overcoming T Cell Exhaustion or Suppression

Complete T cell activation requires costimulatory signals, typically provided by APCs and cancerous cells in the TME. However, the TME downregulates the expression of costimulatory molecules in cancerous cells as a mechanism to avoid immune detection. Furthermore, the TME also upregulates coinhibitory molecules that suppress T cell activation, further hindering anti-tumor immunity. Consequently, targeting these coinhibitory ligands and their receptors has been a successful immunotherapy [112]. To take advantage of this, OVs can be armed with either costimulatory molecules or with immune checkpoint inhibitors (ICIs) to amplify T cell activation within the TME.

A similar approach involves arming OVs with ICIs (Figure 5C). The inhibitory checkpoint proteins that can be targeted include CTLA-4 (i.e., cytotoxic T-lymphocyte-associated protein 4), PD-1 (i.e., programmed cell death 1), or PD-L1 (i.e., programmed cell death ligand 1). ICI-encoding OVs that produce antibodies against CLTA-4, PD-1, and PD-L1 are more effective than their parental counterparts at eliciting an anti-tumor response [113]. Novel checkpoint protein targets such as TIGIT (i.e., T cell immunoreceptor with immunoglobulin and ITIM domains), TIM-3 (i.e., T cell immunoglobulin and mucin domain-containing protein 3), and BTLA (i.e., B- and T-lymphocyte attenuator) are also under current investigation [79].

One of the most novel engineering methods for OVs involves arming them with bi- or trispecific T cell engager (BiTE or TriTE) molecules (Figure 5D). These molecules replace the necessity for APCs in T cell activation. BiTEs are recombinant proteins composed of two linked single-chain fragment variables sourced from two different antibodies, with one fragment targeting a T cell co-receptor molecule, such as CD3, and the other targeting tumor-specific antigens [114]. TriTEs differ as they target three proteins [114]. Wang et al. demonstrated that an ADV encoding a BiTE could amplify anti-tumor activity and further increased the number of CTLs in mouse models [115]. The ability of this strategy to precisely augment T cell responses against tumor-associated antigens makes it a highly attractive approach for boosting the OV efficacy against specific cancers.

A final strategy to enhance anti-tumor immune responses involves using OVs as cancer vaccines. When OVs infect tumor cells, they trigger inflammatory responses directed against viral proteins. Therefore, in turn, they can elicit an immune reaction against tumor-associated antigens, even without prior knowledge of specific antigenic targets [116]. This concept underlies cancer vaccines, which exploit the inherent immunogenicity of viruses to activate anti-tumor adaptive immune responses [116]. In situ vaccination with OVs can lead to systemic responses which could be effective against metastatic disease [117]. Modifying viruses to enhance their ability to trigger immune responses against specific or unknown TAAs represents a promising approach to generate robust systemic anti-cancer immunity [103].

## 5. The Current Landscape of Oncolytic Virotherapy for Uveal Melanoma and Retinoblastoma

While no OVs are currently approved for ocular tumor treatment, significant breakthroughs have been achieved in other cancers, particularly cutaneous melanoma. T-VEC, approved by the FDA in 2015 for advanced cutaneous melanoma, marked the first OV therapy. OVs are a great alternative to conventional therapy, with lower side effects when compared to radiation therapy, mainly in preserving the macula. Although OV treatments for ocular tumors have shown promise in preclinical studies, clinical translation remains a challenge with limited clinical trials for OV-based therapies for UM and RB (Table 4). This section will review the current landscape of OV therapy in both UM and RB.

### 5.1. HSV-Based Oncolytic Viruses

In regard to UM, Liu et al. examined the combined administration of oncolytic HSV1 and toll-like receptor 3 (TLR3) [121]. This combination therapy stimulated an immune response mediated by macrophages and enhanced anti-tumor activity in UM cell lines, suggesting its potential for continued investigation [121]. Another study demonstrated that arming HSV-1 to encode GM-CSF led to a more significant reduction in the UM tumor volume in xenograft mouse models compared to unmodified HSV-1 [122]. Further supporting the potential of HSV-1 based therapies, researchers demonstrated that HSV-1 treatment reduced intraocular tumors by increasing anti-tumorigenic M1 macrophages, while decreasing pro-tumorigenic M2 macrophages. They also observed an increased infiltration of NK cells and mature dendritic cells, indicating a robust immune response induced by HSV-1 therapy [123]. One study showcased a novel strategy to eliminate UM using HSV-1-encoding E. coli cytosine deaminase to deliver the prodrug 5-fluorocytosine. In infected UM cells and in vivo xenograft models, the HSV-1 OV-delivered cytosine deaminase converted 5-fluorocytosine into 5-fluorouracil, a potent anti-cancer drug, resulting in decreased tumor volumes and increased survival [124]. This study highlights a new approach of using OVs to precisely deliver and activate potent anti-tumor drugs. Despite the promise of these preclinical studies, no HSV-1-based therapies are currently being evaluated in a clinical setting for the treatment of UM.

Several approaches have been employed to harness the oncolytic potential of the HSV for the treatment of RB. One strategy involves modifying the HSV by deleting the ribonucleotide reductase gene, resulting in selective viral replication in rapidly dividing cells, such as cancer cells. This modified HSV was examined as a potential oncolytic virotherapy in murine models of RB; however, intraocular injections of the modified virus did not result in either replication nor anti-tumor effects in these murine RB models [125]. Another study engineered the HSV to express thymidine kinase and modified genetic components of the virus to allow for selective replication in RB cells. The administration of this modified HSV with ganciclovir in RB cell lines led to tumor-specific ganciclovir phosphorylation, which inhibits DNA synthesis and causes ICD in tumor cells [126]. These studies highlight the potential of a genetically modified HSV to induce oncolysis in RB, but further research is needed prior to clinical translation.

### 5.2. ADV-Based Oncolytic Viruses

ICOVIR-5 is an enhanced ADV with increased tumor-selective expression and replication [127]. It achieves this by modifying its genetic elements to allow for the selective replication of the virus in tumors with deregulated E2F–retinoblastoma pathways [127]. A phase I clinical trial completed in 2023 examined the administration of IVOCIR-5 in both cutaneous and uveal melanoma with limited success [119]. The study found that although ICOVIR-5 could reach melanoma metastases after a single intravenous dose, there was no regression of tumors [119]. A series of studies have also explored an alternative ADV-based therapy for UM. Cun et al. investigated the effects of combining ADV, H101, and alkylating agent dacarbazine in in vitro models of UM, demonstrating a synergistic effect in promoting UM ICD [128]. Another study showed that combining H101 with small interfering RNA (siGNAQ) could induce apoptosis in UM cell lines harboring GNAQ mutations [129]. These preclinical results support the continued study of H101 as a treatment for UM.

ADVs have emerged as the most widely studied OVs for RB. Initial studies attempted to modify ADV to deliver HSV tyrosine kinase to tumors. Although this strategy could inhibit RB cell growth in vitro, intravitreal injection in vivo elicited only a mild immune response [126,130]. Furthermore, H101 inhibited RB cell growth in both in vitro and mouse xenograft models, demonstrating its potential therapeutic use [131]. Meanwhile, Song et al. demonstrated that combining ADV (SG600) with vincristine, a drug that inhibits microtubule formation, had a synergistic effect in in vitro studies [132]. Despite the promise of some of these studies, none have progressed into the clinical setting. By far the most promising studies have been with VCN-01, an adenovirus modified to selectively replicate in cells with an abundance of free E2F-1, a hallmark of RB [72]. In xenograft mouse models, the intravenous administration of VCN-01 induced RB necrosis and vitreous inflammation and prevented disease dissemination [72]. These results led to the initiation of a phase I clinical trial (NCT0328268) that is examining VCN-01 in patients with refractory RB.

### 5.3. RNA-Based Oncolytic Viruses

Researchers are also exploring RNA viruses as potential therapeutics for UM and RB. Notably, a VSV armed to express IFNβ can induce the oncolysis of tumor cells and initiate a robust immune response associated with the recruitment of cytotoxic CD8^+^ T cells, which prevented tumor growth in animal models [133]. Additionally, this effect can be augmented by combining this oncolytic virotherapy with ICIs [133]. This study has led to a current phase I clinical trial which is examining VSV-expressing human IFNβ and tyrosinase-related protein 1 (TYRP-1) in patients with UM (NCT03865212). The results from this phase I trial have demonstrated that VSV-IFNβ-TYRP1 can be safely administered via both intratumoral and IV routes in patients with UM, leading to immunogenicity, although no notable radiographic response has been observed [90]. The researchers propose to combine VSV-IFNβ-TYRP1 with additional target antigens and immunomodulatory treatments to avoid immune escape in future trials [90]. Coxsackievirus has also been examined in patients with metastatic UM. In a phase Ib clinical trial (the CLEVER study) coxsackievirus A21 was administered in combination with ipilimumab, an antibody against CTLA-4 (NCT03408587). Patients receiving the combination therapy had a manageable safety profile; however, 8 out of 11 patients had progressive disease [120]. While many studies such as the CLEVER study have demonstrated the safe administration of OVs in metastatic UM, achieving promising clinical results, specifically tumor regression, remains a challenge.

These diverse strategies highlight the potential of OV therapies for metastatic UM, with ongoing efforts focused on enhancing the tumor specificity and stimulating anti-tumor immunity. However, translating these promising preclinical findings into clinical success and improved patient outcomes remains a crucial challenge for future research.

Conversely, the only notable RNA-based OV that has been studied as a therapy for RB is the SVV. Two studies assessed SVV-001 in both in vitro RB cell lines and xenograft RB murine models, demonstrating that SVV-001 is highly selective to RB cells with high oncolytic properties that also prevent disease progression in murine models [134,135]. These studies led to a phase I clinical trial (NCT03865212) which investigated an SVV-based OV. Although well tolerated in pediatric patients with neuroendocrine tumors, including RB, NTX-010 demonstrated limited efficacy when used alone or in combination with cyclophosphamide, potentially due to the presence of neutralizing antibodies [136].

Overall, these preclinical and clinical studies underscore the potential of oncolytic virotherapy for the treatment of RB and UM. Despite the challenges encountered, the diverse approaches under investigation offer hope for the development of effective OV therapies. Further research is warranted to translate these promising findings into clinical success and ultimately improve treatment outcomes for patients with RB.

### 5.4. Virus-like Drug Conjugates

Although VDCs do not function as OVs mechanistically, it is important to underscore the recent advances in the literature regarding the use of AU-011 for the treatment of UM. AU-011, also known as belzupacap sarotalocan, consists of a human papilloma virus (HPV)-derived virus-like particle (VLP) loaded with a cytotoxic drug and coupled with approximately 200 molecules of phthalocyanine dye [137]. Upon light activation, the VDC binds to heparan sulfate proteoglycans (HSPGs) on tumor cells, subsequently leading to tumor cell necrosis through oxidative stress or immune-mediated tumor cell killing through the damage-associated molecular patterns (DAMPs) pathway.

Currently under investigation in a phase III clinical trial (NCT06007690), previous results showed that a subchoroidal injection of AU-011 was effective in inducing tumor regression in a rabbit model of ocular melanoma [138,139]. In humans, the phase 1b/2 results showed that the intravitreal administration of AU-011 led to great local tumor control with good tolerance [140,141]. Similarly, preliminary results regarding the subchoroidal administration of AU-011 showed a positive safety profile, with the main reported adverse effects being anterior chamber inflammation, conjunctival hyperemia, punctate keratitis, and eye pain [142].

## 6. Immunotherapy

The introduction of immunotherapy made a big impact in cancer therapy by bolstering the immune system’s ability to target and eliminate cancerous cells. Often, the progression of cancer is directly linked to the state of the immune system. Cancerous cells can evade the natural defenses of the immune system, enabling them to continue proliferating. However, if the tumor is not eliminated, the cancerous cells eventually overwhelm the immune system due to immune exhaustion or by suppressing the immune system [143]. There are many different biomolecular pathways that drugs may target to bolster the immune response against proliferating cancerous cells. In this section, we will describe the different pathways used for the treatment of intraocular tumors, including ICIs, which attempt to overcome T cell exhaustion, and cell-based therapies that enhance the anti-tumor functions of patient-derived T cells [144,145].

### 6.1. Immune Checkpoint Inhibitors

**Figure 7:**
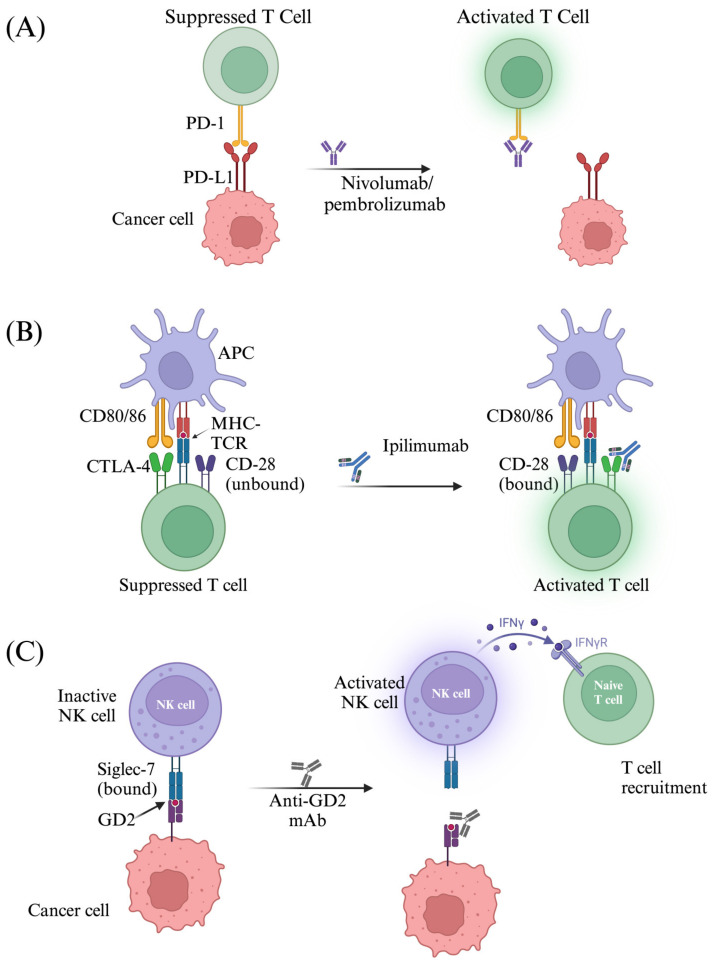
A schematic illustration of the mechanism of action of immune checkpoint inhibitors. (**A**) Nivolumab and pembrolizumab block the PD-1 receptor, interfering with PD-L1 mediated immunosuppression. (**B**) Ipilimumab binds CTLA-4, which inhibits it from competing with CD-28 for CD80/86, leading to increased costimulatory T cell signaling. (**C**) Anti-GD2 antibodies block tumor-expressed GD2 from interacting with its receptor, Siglec-7, restoring NK cell function. Created in BioRender. Tuli, N. (2025) https://BioRender.com/b97r111.

Immune checkpoint inhibitors (ICIs) represent a groundbreaking class of cancer therapies that harness the body’s immune system to target and eliminate cancer cells. By inhibiting specific proteins, such as PD-1, PD-L1, and CTLA-4, which ordinarily suppress immune responses, these therapies restore the ability of T cells to recognize and attack tumors [146] (Figure 7). ICIs have revolutionized oncology, showing remarkable efficacy across various cancer types. Currently, several ICIs are approved for clinical use in non-retinal tumors, including nivolumab and pembrolizumab, both targeting PD-1 [147,148]. Additionally, ipilimumab, an anti-CTLA-4 therapy, has been approved, particularly in combination with other ICIs for cancers such as cutaneous melanomas [149].

#### 6.1.1. Targeting PD-1/PD-L1 Interaction

PD-1, a cell surface receptor expressed on many immune cells, most notably tumor-infiltrating T cells, is an immune checkpoint protein involved in the induction of apoptosis upon binding its ligand, PD-L1 [150]. While crucial for regulating excessive or harmful immune responses, this mechanism can be exploited by tumors to evade immune surveillance and promote growth. PD-L1 exhibits pro-tumorigenic properties by inducing proliferation and preventing programmed cell death. Tumors can attenuate the host’s immune response by upregulating their PD-L1 expression, which will inhibit T cell activation through PD-1, therefore inhibiting anti-tumor immune responses. Tumor cells exploit the PD-1/PD-L1 interaction to suppress immune responses and evade destruction. Disrupting this interaction with ICIs, such as the monoclonal antibodies (mAbs) pembrolizumab and nivolumab, can reinvigorate anti-tumor immunity. These antibodies bind to PD-1 receptors on T cells, blocking the interaction with PD-L1 expressed on tumor cells (Figure 7A) [147,148]. This blockade releases the brakes on T cell activity, enabling them to recognize and eliminate cancer cells more effectively. The reactivated T cells then release cytokines and other immune mediators, amplifying the anti-tumor immune response [151].

#### 6.1.2. CTLA-4

CTLA-4 represents another target for ICIs. Like PD-1, CTLA-4 is an inhibitory receptor found predominantly on T cells which downregulates T cell receptor signaling to inhibit T cell activation. It competes with CD28, a costimulatory molecule, to bind B7 (CD80/86) receptors on APCs, ultimately inhibiting T cell cytokine production and inducing cell cycle arrest [152]. The balance between CD28 and CTLA-4 signaling is a key regulator of T cell activation, with the former promoting activation and the latter inhibiting it. A notable difference between CTLA-4 and CD28 is that CTLA-4 is antigen-independent and suppresses T cell activation even in the absence of TCR signaling [153]. Regulatory T cells (Tregs) constitutively express CTLA-4 to prevent excess inflammation and attenuate immune responses [153]. Tregs, through CTLA-4 pathways, can downregulate B7 expression by APCs, therefore reducing CD28 stimulation and subsequent T cell activation [153]. CTLA-4 is overexpressed by tumor-exhausted T cells, infiltrating Tregs, and even tumor cells themselves [154,155]. Tumors can also lower the CD80/86 costimulatory molecules expressed by APCs, which can deplete the TME of costimulatory molecules, thus raising the threshold for T cell activation [156]. ICIs targeting CTLA-4, such as ipilimumab, work by blocking the interaction between CTLA-4 and its ligands (B7-1 and B7-2), thereby preventing T cell suppression (Figure 7B). This blockade restores immune function by promoting T cell activation and proliferation, enhancing the ability of cytotoxic T cells to recognize and destroy tumor cells [157]. Additionally, a CTLA-4 blockade can deplete immunosuppressive Tregs, further amplifying the anti-tumor response [158]. Ultimately, CTLA-4 ICIs reprogram the TME, shifting it from a state of immune suppression to one of immune activation, allowing the body to mount a more effective attack on cancer cells.

### 6.2. Adoptive Cell Transfer Therapy

**Figure 8:**
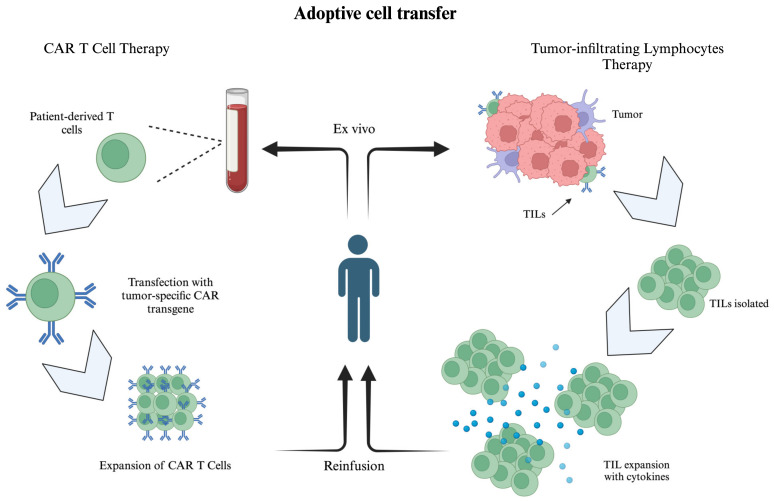
A schematic of adoptive cell transfer therapies. LEFT: A schematic of CAR T cell therapy, where patient-derived T cells are transfected with tumor-specific CAR transgenes, leading to the expression of CARs. These cells are then expanded and re-infused into the patient. RIGHT: A schematic of TIL therapy whereby TILs are isolated from a patient-derived tumor specimen, isolated, and then expanded with cytokines ex vivo. The TILs are then re-infused into the patient. Created in BioRender. Kulbay, M. (2025) https://BioRender.com/x32x257.

ACT therapies, such as tumor-infiltrating lymphocyte (TIL) and chimeric antigen receptor T cell (CAR T) therapies, have emerged as promising therapies in the field of oncology (Figure 8). These therapies rely on exploiting and bolstering the patient’s own existing tumor-specific T cell population to increase subsequent immune responses [159]. A challenge of TIL therapy is the need to disrupt the immunosuppressive equilibrium within the system, which is inherently designed to counteract the changes induced by ACT. For this reason, TIL therapy is often coupled with ICIs, in order to have an effect on immune modulators like T-regulation cells [160]. While CAR T cell immunotherapy was traditionally employed for blood cancers, including a hugely successful application of anti-CD19 CAR T cells to treat B-cell malignancies like leukemia, there is a shift towards using the technology for solid tumors [161,162]. The difficulty of transferring to solid tumors is the difference in tumor pathways, namely in finding a suitable target antigen that would not have an adverse effect that is too impactful on the host [162]. Furthermore, CAR T cells have increased difficulty in penetrating solid tumors [163]. It is important to remember that some receptors that appear on cancerous cells also appear on normal tissue. As such, a CAR T cell must be developed with tumor-specific CAR receptors so the modified T cells can differentiate cancerous tissue from normal tissue.

### 6.3. T Cell Receptor-Bispecific Molecules

The melanoma-associated antigen, known as gp100, may be used as a pathway to redirect cytotoxic T cells towards cancer cells [164]. The gp100 peptide, found on melanoma cells, is presented by HLA-A*02:01. MHC/HLA are important facets of the immune system, since they enable the presentation of small peptides which are then recognized by TCRs and, upon recognition by T cells, lead to their activation and anti-tumor effects [165]. However, gp100 presented by HLA-A*02:01 is not naturally recognized by TCRs, so it is not naturally used as a target protein for T cell activation. Tebentafusp was engineered to address this issue. Tebentafusp is a TCR-bispecific molecule, composed of a fusion of a high-affinity TCR β chain with a humanized CD3-specific single-chain antibody fragment [166]. In other words, tebentafusp is a bispecific fusion protein with two domains, one anti-CD3 domain that has an affinity for T cells and the other domain that recognizes the gp100 peptide of HLA-A*02:01 on melanoma cells [167]. When tebentafusp is introduced into the tumors, it facilitates the interaction between T cells and the gp100 antigen expressed by melanoma cells. Therefore, it bridges the gap between the cells presenting gp100 and the T cells that normally struggle to recognize the peptide. These T cells then become activated, inducing cytotoxic death in gp100-expressing tumor cells. Thus, tebentafusp can increase the system’s repertoire of cancer-reactive T cells. However, it is important to note that this pathway only exists in individuals expressing HLA-A*02:01. According to population studies, the frequency of HLA-A*02:01 can vary between 11.9% to 27.1%, depending on ethnicity [168]. Therefore, it is limited to a small proportion of affected individuals, reducing the potential impact of this drug. Since gp100 is a melanocyte marker, this pathway is not a relevant treatment for RB.

### 6.4. Targeted Immunotherapy

A more novel approach in the treatment of intraocular tumors involves the use of targeted immunotherapy. Conversely to immunotherapy, targeted immunotherapy directly impacts the tumor growth, survival, and/or spread via the direct regulation of tumor-related cellular and/or biomolecular signaling pathway partners, as discussed in Section 2 [169]. Recently, these agents have shown promising results. However, side effects of greater importance such as uveal effusion, retinal detachments, and retinopathies have also been reported [170,171]. Systemic side effects with targeted immunotherapy have also been reported, such as kidney failure and hepatitis. In those cases, which represent a small proportion of patients, the clinical decision to pursue targeted immunotherapy must be carefully reconsidered. Despite these challenges in their clinical application, they remain a great alternative for the treatment of UM and RB. In this section, we review the most common targets for targeted immunotherapy applied for the treatment of UM and RB.

#### 6.4.1. B7-H3 Protein

While CTLA-4 and PD-1 have been the stars of ICIs, researchers have identified other immune checkpoint molecules potentially linked to retinal tumor proliferation, notably B7 homolog 3 protein (B7-H3). This molecule exhibits significantly increased expression in RB tumor cells [172]. B7-H3 is an inhibitory checkpoint molecule similar to CTLA-4 and PD-1 that has been studied in the context of auto-immune diseases and cancer, where it was shown to be a key inhibitor of T cell proliferation [173]. B7-H3 has been found to be irregularly upregulated [173,174], and high levels of tumor-expressed B7-H3 were shown to be associated with a poor prognosis in various cancers, such as laryngeal squamous cell carcinoma [175], non-small cell lung carcinoma [176,177], colorectal carcinoma [178], and RB [172]. Despite being highly associated with a poor cancer prognosis and known to have an immunosuppressive function, the exact receptor of B7-H3 has not yet been identified. However, targeting B7-H3 with monoclonal antibodies (mAbs), akin to other ICIs, may be a viable therapeutic strategy to restore T cell activation and proliferation.

#### 6.4.2. GD-2

GD2 is a disialoganglioside that is expressed in certain cancer cells and has been observed in many pediatric cancers, including RB [179]. Interestingly, GD2 is also present on melanocytic tumor cells, including in UM [180]. While many gangliosides are not viable targets for treatment because of their widespread expression on normal tissue, there are certain gangliosides that are specific to cancer cells [181]. GD2 is considered a tumor-associated antigen and may be a suitable target [182]. GD2 expression has been found to be associated with many pro-tumorigenic features, such as the downregulation of the immune system and angiogenesis [183]. GD2 acts on immune checkpoint receptor Siglec-7, suppressing immune cells and more predominantly NK cells (Figure 7C) [184]. However, some studies have shown that the overexpression of GD2 may be linked to a poor cancer prognosis [183], such as in breast cancer [185,186]. The prognostic value of GD2 in RB has been limited and reported in only a few studies previously [187,188]. The development of mAbs specific to GD2 is currently underway, as researchers are hoping to counteract GD-2-mediated immunosuppression, which include three pathways to destroy tumor cells expressing GD2: (i) the induction of phagocytosis by macrophages, (ii) lysis through complement-dependent cytotoxicity, and (iii) the direct induction of death via the binding of mAbs to GD2 [182].

#### 6.4.3. Gα Signaling Pathway

As previously discussed, abnormalities within the Gα signaling pathway are present in the majority of UM cases due to activating mutations in the *GNAQ*/*GNA11* genes. The constitutive activation of these genes leads to tumor cell proliferation and survival through various signaling pathways (Figure 1). The selective targeting of each Gα signaling pathway partner has been thoroughly reviewed recently and we suggest referring to that review for further details [189]. Although great interest has been shown in targeting the Gα signaling pathway over the past few years, the efficiency of these agents is yet to be fully elucidated when compared to ICIs.

## 7. The Current Landscape of Immunotherapy and Targeted Immunotherapy for Uveal Melanoma and Retinoblastoma

Immunotherapies have emerged as a promising avenue for treating UM and RB, offering potential benefits over traditional therapies. This section will review the most recent preclinical studies and the ongoing clinical trials investigating immunotherapies in UM and RB (Table 5), specifically focusing on the advancements in ICIs and ACT therapies. By understanding the current research landscape, we can better assess the potential of these innovative approaches to improve treatment outcomes and advance the field of ocular oncology.

### 7.1. Novel Treatments for Uveal Melanoma

Several ICIs, including ipilimumab, are currently approved by the FDA for treating cutaneous melanoma [152]. Since this is a long-established drug and biomedical pathway, there are limited recent preclinical trials, and most of the focus is on clinical trials for metastatic UM. Completed clinical trials have shown limited efficacy with an indication of the immune-related control of the disease. In a phase I study by Maio et al., 82 patients with metastatic UM received ipilimumab, and they found a weak response, with 34% of patients having immune-stable disease for at least 3 months, and the reported overall median survival was 6 months [198]. For this reason, there are no current clinical trials testing ipilimumab alone for the treatment of metastatic UM. However, there are several ongoing clinical trials examining the use of ipilimumab in combination with other ICIs, ACT therapies, or chemotherapies. Another anti-CTLA-4 drug that has been gaining traction in recent clinical trials is RP2, an anti-CTLA-4 antibody-like molecule that can act as an ICI [199]. There are currently two clinical trials involving RP2 as a treatment for metastatic UM, in combination with nivolumab. Despite the promising results of ICIs in cutaneous melanomas that have led to FDA approval, the application of ICIs in metastatic UM remains to be developed.

In the case of metastatic melanoma, combining ipilimumab with nivolumab or pembrolizumab has shown an increased efficacy compared to groups taking only one set of drugs [200]. A study by Larkin et al. found that in a cohort of 945 patients with metastatic melanoma, including metastatic UM, the median progression-free survival rate was 11.5 months for the group receiving the combined treatment, compared to 2.9 months with only ipilimumab and 6.9 months with only nivolumab [201]. However, in the case of metastatic UM, the difference was smaller, but still noticeable. Another study by Pelster et al. found that the treatment of metastatic UM with a combination of ipilimumab and nivolumab in a cohort of 33 patients resulted in an overall response rate of 18% and a median progression-free survival rate of 5.5 months, leading them to conclude that combined treatment leads to a response from the immune system that is evident and sustained, although 40% of patients experienced a treatment-related adverse event [196]. There are currently nine clinical trials for metastatic UM that include a combination of these two drugs in the treatment plan. These drugs are also being studied in combination with radiotherapy, chemotherapy, targeted therapy, or vaccine therapies.

A preclinical study conducted by Harper et al. demonstrated that tebentafusp increased the T cell anti-tumor activity, leading to increased tumor lysis without significant adverse effects, in their cell line model [202]. A study by Middleton et al. with 84 patients with advanced melanomas, including metastatic UM, found that treatment with tebentafusp results in a 65% 1-year survival rate and, interestingly, demonstrated that the tebentafusp intake was correlated with an upregulation of cytotoxic T cells in the TME [164]. It is important to note, however, that only patients possessing the HLA-A*02:01 haplotype can benefit from tebentasfusp, limiting the generalizability of the treatment.

An alternative to immunotherapy is the use of targeted immunotherapy, where treatment modalities aim to decrease the uncontrolled activation of the signaling pathway involved in UM (Figure 1) [189]. Darovasertib, a protein kinase C (PKC) inhibitor, was approved by the FDA in 2022 for the treatment of metastatic UM [203]. A phase 2 clinical trial, aiming to establish darovasertib neoadjuvant/adjuvant therapy’s efficacy and safety in localized UM, showed that neoadjuvant therapy induced tumor shrinkage, with non-serious adverse effects such as postural hypotension, syncope, rash, pruritus, fatigue, diarrhea, nausea, and vomiting [204]. Additional targeted immunotherapies are summarized in Table 6. Finally, there are several ongoing clinical trials examining ACT therapies for metastatic UM, including both TIL and CAR T cell therapies. In a recent preclinical study, Gezgin et al. tested the feasibility of obtaining TILs from metastatic UM patients to use as an adjuvant immunotherapy for at-risk metastasis patients. They found that it was a viable strategy because natural T cells reactive to UM are suppressed within the TME [205]. In essence, there are several TIL-based clinical studies for metastatic UM, with some studies examining TILs alone, whereas others combine TILs with ICIs and other oncologic agents. Aside from TIL therapies, there is also significant interest in CAR T cell therapies, although they are at an earlier stage of development. As this is a newer development in immunotherapy, it may take more time for more clinical trials involving CAR T cell therapy to appear, especially since there have been some difficulties in translating its success to solid tumors in preclinical trials [162].

### 7.2. Retinoblastoma

While there have historically been many avenues of research of immunotherapies for metastatic cutaneous melanoma, there is limited research on this approach for RB due to many factors including the high level of efficacy of other treatments like chemotherapy and focal therapies [47]. Nonetheless, even if RB is a treatable disease if found in the early stages, it poses a great risk to the patient if undiagnosed. In these situations, it would be useful to find alternative treatment options that do not rely on invasive surgeries or enucleation.

One of the more promising targets for RB immunotherapy is GD-2, which has been targeted with mAbs and CAR T cells [180]. One study demonstrated that GD-2-specific mAbs can cause significant tumor lysis in RB cell lines, demonstrating possible cell-mediated cytotoxicity caused by the mAbs [206]. In the same study by Eichholz et al., four patients with stage IV RB were treated with an anti-GD2 mAb, Dintuximab beta [206]. The clinical arm of the study found that Dintuximab beta treatment in four patients with metastatic RB may have helped in prolonging the remission of the disease [206]. Alternatively, in another study, Sujjitjoon et al. investigated GD2-specific CAR T cell therapy for RB [207]. While these CAR T cells were effective in destroying tumorous RB cells in vitro at first, the cancer built a resistance to them, and there was an observed upregulation of PD-L1 expression [207]. Therefore, the authors suggested that this treatment may not be adequate on its own and should be combined with other therapies to be effective [207]. As this is a relatively new field, there are currently no ongoing clinical trials investigating GD-2-specific therapies against RB.

While GD2 remains a promising target, clinical studies for RB are increasingly focusing on CAR T cell therapy targeting B7H3 and EGFR806. In preclinical studies, researchers found that B7-H3 inhibits the cytotoxic activity of NK cells and T cells against tumor cells [173]. B7-H3 expression has also been found to be a negative prognostic factor in cancer [208,209]. Thus, currently, a phase I trial is being conducted on RB patients using B7-H3 CAR T cells and pembrolizumab (NCT04483778). While B7-H3 continues to be investigated, EGFR806-specific T cells are also being studied. EGFR806 is upregulated in glioblastomas and other solid central nervous system tumors, including RB [210]. In preclinical studies, it was demonstrated that EGFR806 CAR T cells could effectively eliminate solid CNS tumors in xenograft mouse models [210]. These promising studies have led to a phase I clinical trial (NCT03618381) investigating EGFR806 CAR T cells for solid tumors, including RB. These two clinical trials investigating CAR T cells are the only trials investigating immunotherapy for RB but offer promising therapies for patients with refractory RB.

In summary, while immunotherapies for UM have shown a slight increase in the average survival times, providing future hope for patients, neoadjuvant treatment for advanced UM and combination treatment modalities remain in the early stages of development. ICIs like ipilimumab and nivolumab have demonstrated limited success in metastatic UM, particularly in combination therapies, though breakthroughs are still awaited. Tebentafusp and ACTs such as CAR T cells offer potential therapeutic responses but require further clinical validation. For RB, immunotherapy research is nascent due to the effectiveness of existing treatments, though new targets like GD-2 and B7-H3 show potential for patients with refractory disease. Overall, targeted immunotherapy appears to be the most promising approach for advancing ocular oncology.

## 8. Challenges and Future Perspectives

Numerous challenges limit the clinical translation of OVs for the treatment of intraocular tumors [211]. Safety and regulatory aspects are a major obstacle. Although OVs have been reported as being safe, the risk of unintentional transmission from patient or infected material to human contacts is non-negligible given the high replication rate of OVs. The results from a viral surveillance program showed that 8.4% of respondents reported signs and symptoms related to HSV infection following T-VEC treatment amongst their close contacts [212]. In the efforts to limit virus propagation during clinical procedures, guidelines for the storage of OVs and their handling and disposal, as well as incident management, have been proposed [213,214,215]. However, efforts in physicians and other healthcare workers’ training, as well as patient education, must be reinforced. Conversely, the handling and storage of OVs poses another barrier for worldwide clinical translation. Studies have shown that the storage of ADV at temperatures of 30 °C for 2 weeks can significantly decrease the infectious titer, as can multiple freeze–thaw cycles [216]. Current guidelines require the storage of T-VEC at temperatures of −80 °C. This causes a barrier to its implementation in pharmacies, as well as in hospitals with limited resources, given the lack of appropriate freezers. To promote the clinical translation of OVs, numerous steps have to be taken, such as (i) establishing effective measures to limit virus propagation and the infectivity of handlers and close contacts, (ii) providing resources to healthcare facilities and carriers to preserve the cold chain, and (iii) promoting campaigns for public awareness towards OVs and their associated side effects and contagiosity.

Another limitation regarding OV clinical translation for the treatment of intraocular tumors regards the data availability, which is sparse. Further research is needed regarding the dosing requirements for intraocular drug bioavailability; dosing adjustments according to the tumor size or volume, while preserving the safety profile, are required. Recent studies have demonstrated the clinical efficiency of OVs for the treatment of metastatic UM as well as for a few cases of RB when administered intravenously. However, IV injections pose a greater threat for widespread OV infection, as well as the viral-induced mutagenesis of normal cells. Further studies regarding the intravitreal administration of OVs are required, mainly in cases with stable systemic disease with progressing vision loss due to tumor metastasis. A challenge remains in terms of managing the adverse effects of these novel treatments. Although the common side effects of OVs are self-limiting, such as fatigue, chills, flu-like symptoms, nausea, and fever [217], more serious complications can arise, such as vasculitis, breathing difficulties, and systemic infection. Given the immunocompromised state of cancer patients, OV administration must be thought through carefully. Clinicians need to establish an adequate dosing treatment to preserve visual function while obtaining adequate control over the tumor growth and limiting recurrence rates.

One of the main challenges involved in targeted immunotherapy involves the treatment of hepatic metastasis from UMs due to hepatic tropism. The hepatic metastic niche has been shown to facilitate UM metastatic colonization [218] due to T cell inactivation, immune tolerance, and cell destruction, which alter the liver sinusoids’ role; it is hypothesized that the liver increases the sensitivity to tumor antigens [219]. Therefore, efforts need to be deployed to elucidate the mechanisms of action to better target liver metastasis. Although pharmaceutical advances in immunotherapy for the treatment of primary and metastatic intraocular tumors have seen significant growth over the past few years, further studies to better understand the role of the TME in tumorigenesis need to be conducted. A better understanding of pathophysiological abnormalities will enable the development of greater treatment tools.

Overall, the management of intraocular tumors involves a personalized approach. Each treatment modality which aims to limit disease progression and/or recurrence comes with its specific burden of side effects and psychosocial impacts. The possibility to limit enucleation or exenteration rates in patients with intraocular tumors with the use of OVs and/or targeted immunotherapy would significantly increase the well-being of patients and increase their quality of life. Numerous efforts have to be deployed in research to establish efficient and safe therapeutic approaches for the treatment of UM and RB.

## Figures and Tables

**Table 1:** Overview of current therapeutic approaches for uveal melanoma.

Therapeutic Approach		Method	Indication	Disadvantages	References
Local Resection		Complete or partial tumor removal through endoresection (ab interno) or transcleration/exoresection (ab externo)	Nasal tumor location: Distance from fovea > 4 mm Distance from optic disc > 3 mm Larger tumors with small basal diameters: Thickness > 8 mm Diameter < 15 mm	Procedural difficulty Risk of severe postoperative complications (vitreous hemorrhage, retinal detachment, and cataracts) Risk of extrascleral or systemic dissemination	[15,19,20,21,22,23]
Enucleation		Complete removal of the globe	Large tumor size: Thickness > 12 mm Diameter > 18 mm A total circumferential tumor invasion around the optic nerve Uncontrollable melanoma-related glaucoma Blind, painful eye	Psychosocial challenges caused by loss of globe	[15,17,18,19,24,25]
Exenteration		Removal of the globe and orbital structures (e.g., muscles, fat, nerves, and the eyelid)	Extraocular tumor growth Polyfocal or recurrent disease in the orbit	Psychosocial challenges caused by the loss of the orbital structures and globe	[15,24,25]
Radiotherapy	Plaque brachytherapy	A radioactive plaque (e.g., Iodine-125) is surgically placed on the episclera overlying the tumor, facilitating the transmission of radiation to the malignancy locally	Small and medium tumor sizes	Several radiation-associated complications can occur: Radiation-induced cataracts Vitreous hemorrhage Neovascular glaucoma Retinal detachment Radiation retinopathy, maculopathy Optic neuropathy	[15,17,18]
	Proton beam radiotherapy	The delivery of a precisely controlled proton beam to the malignancy; tantalum markers ensure that a homogenous radiation dose is applied to the entire tumor	Small- to large-sized tumors	Several radiation-associated complications can occur: Radiation-induced cataracts Vitreous hemorrhage Neovascular glaucoma Retinal detachment Radiation retinopathy, maculopathy Optic neuropathy Changes in eyelid and eyelashes High costs and limited availability	[15,18,26]
Laser Therapies	Transpupillary thermotherapy	Delivery of heat to tumor tissue to promote cell death through infrared diode laser	Small-sized tumors (<3 mm thickness), tumors in the posterior pole	Tumor recurrence Complications include the following: Branch retinal vascular obstruction Retinal traction and tractional retinal detachment Macular edema Neovascularization of the retina Retinal hole Optic disc edema Focal iris atrophy Posterior synechiae	[19,27]
	Photodynamic therapy	Selective destruction of cancer cells by reactive oxygen species generated through light-activated photosensitizers	Small (<3 mm thickness) tumors in the posterior pole	Tumor recurrence Complications include the following: Post-procedural pain Inflammation Vitreous hemorrhage Retinal pigment epithelium atrophy	[28,29,30,31]

**Table 2:** Overview of current therapeutic approaches for retinoblastoma.

**Therapeutic Approach**		**Method**	**Indication**	**Disadvantages**	**References**
Enucleation		Complete removal of the globe	Primary enucleation: Advanced unilateral retinoblastoma without salvageable vision Unilateral retinoblastoma with diffuse anterior segment chamber involvement Unilateral retinoblastoma with total vitreous hemorrhage Unilateral retinoblastoma with accompanying neovascular glaucoma Retinoblastoma with a phthisical eye Secondary enucleation: Recurrent retinal tumor not controlled with current treatment strategies Presence of vitreous seeds or subretinal seeds with limited response to chemotherapy and/or radiotherapy Opaque media (total hyphema or dense vitreous hemorrhage) after initial chemotherapy and/or radiotherapy treatment	Psychosocial challenges caused by loss of globe	[35,38,39,40]
Exenteration		Removal of the globe and orbital structures (e.g., muscles, fat, nerves, and the eyelid)	Extraocular involvement and proptosis Recurrent orbital mass tumor after enucleation Typically reserved for cases of advanced retinoblastoma where chemotherapy and/or enucleation did not limit tumor spread to orbital structures	Psychosocial challenges caused by loss of globe and orbit	[39,41,42,43]
Chemotherapy	Systemic Intravenous Chemotherapy	Intravenous administration of chemotherapy drugs that circulate throughout the body	Bilaterial retinoblastoma After the enucleation of the globe with high-risk features for metastasis When delivery of IAC is challenging (e.g., for patients aged under 4 months)	Complications include the following: Hearing loss Growth abnormalities Transient vincristine-related neuropathy Neutropenia Anemia Thrombocytopenia	[32,36]
	Intra-Arterial Chemotherapy	Direct infusion of chemotherapy drugs into the ophthalmic artery through catheterization	Primary treatment: Unilateral retinoblastoma Bilateral retinoblastoma Secondary treatment: Recurrent or persistent tumor/vitreous or subretinal seeds after systemic chemotherapy	Procedural difficulty and endovascular expertise Requirement of specialized facilities Inability to target metastasizing cancer Risk of vascular toxicity Financial cost Complications include the following: Vitreous hemorrhage Hyperemia Ptosis Focal madarosis Forehead erythema Contraindications include presence of neovascular glaucoma, hyphema, vitreous hemorrhage, aseptic preseptal or orbital cellulitis, extraorbital involvement, scleral or optic nerve extension, and trilateral retinoblastoma	[36,44,45,46]
	Intravitreous Chemotherapy	Injection of chemotherapy drugs directly into the vitreous body using a trans-pars plana needle insertion	Recurrent or non-responsive vitreous seeding	Complications are typically localized, such as retinal pigment mottling Higher doses can be associated with rare, but severe, complications: Phthisis bulbi Hypotony	[32,47,48]
	Periocular Chemotherapy	Injection of chemotherapy drugs into the tissue encompassing the eye, specifically in the subconjunctival or sub-Tenon’s space	Advanced retinoblastoma with subretinal or vitreous seeds requiring a highly concentrated local dose	Complications include the following: Orbital and eyelid edema and ecchymosis Orbital fat atrophy Strabismus related to muscle fibrosis Optic atrophy	[32,36,49]
	Intracameral Chemotherapy	Injection of chemotherapy drugs directly into the anterior chamber	Advanced retinoblastoma with aqueous seeding	Procedural difficulty and technical expertise required Complications include the following: Cataracts Iris atrophy Posterior synechiae	[32,36,50,51]
Radiation Therapy	Plaque Brachytherapy	A radioactive plaque (e.g., Iodine-125) is surgically placed on the episclera overlying the tumor, facilitating the transmission of local radiation to the malignancy	Secondary treatment for chemo-resistant, residual macular, and medium-sized tumors (<16 mm basal diameter and >3 mm to <9 mm thickness)	Several radiation-associated complications can occur: Radiation-induced cataracts Vitreous hemorrhage Neovascular glaucoma Retinal detachment Radiation retinopathy, maculopathy Optic neuropathy	[47,52,53]
	External Beam Radiotherapy	Delivery of radiation to the eye and surrounding orbit using a linear accelerator	Extraocular tumor extension Orbital recurrence Positive optic nerve margin post-enucleation Failure of combined chemotherapy and focal therapy	Associated with many complications, including the following: Dry eye syndrome Filamentary keratopathy Cataracts Radiation retinopathy Optic neuropathy Risk of late-onset cancers in children with germline mutation	[35,47,54,55,56,57,58]
Focal Therapy	Transpupillary Thermotherapy	Delivery of heat to tumor tissue to promote cell death through infrared diode laser	Primary treatment: Small tumors (<3 mm thickness) Small, recalcitrant tumors Secondary treatment: Tumors where chemoreduction did not provide satisfactory turmor regression	Complications include the following: Focal iris atrophy Lens opacities Retinal detachment Disc edema	[33,54,59,60,61,62]
	Cryotherapy	Application of extreme cold to freeze and destroy tumor cells using a cryoprobe	Small, peripheral tumors (<3 mm diameter, <2 mm thickness)	Challenges in accurately placing probe for tumors in posterior pole Complications include the following: Vitreous hemorrhage Development of subretinal fluid Retinal holes Contraindications include presence of subretinal fluid	[34,54,63]
	Photocoagulation Laser	Application of laser energy to coagulate and destroy tumor cells	Small, posterior tumors (<3 mm diameter, <2 mm thickness)	Complications include the following: Retinal detachment Vitreoretinal traction Retinal vascular occlusion Retinal holes Preretinal fibrosis Iris burns at the pupillary margin Focal lens opacities Subhyaloid and vitreous hemorrhage Contraindications include presence of vitreous seeding	[33,54,56,64,65]

**Table 4:** Summary of the ongoing and completed clinical trials investigating oncolytic virotherapies for the treatment of uveal melanoma and retinoblastoma.

Clinical Trial Identification (Start Year–End Year)	Phase	Oncolytic Virus Species and Route of Administration	Disease	Study Title Study Conclusion
NCT03284268 (2017–2024)	I	Adenovirous, intravitreal injection	Retinoblastoma	**Evaluate Safety and the Oncolitic Adenovirus VCN-01 Activity in Patients with Refractory Retinoblastoma** VCN-01 demonstrated safety and efficacy in treating retinoblastoma and received an FDA designation for refractory retinoblastoma as an adjunct to chemotherapy [118].
NCT03865212 (2019–2027)	I	Modified Vesicular Stomatitis Virus, single intravenous infusion and injection into liver metastasis	Metastatic Uveal Melanoma	**Modified Virus VSV-IFNbetaTYRP1 in Treating Patients with Stage III-IV Melanoma** The modified VSV can be safely administered intratumorally and intravenously, albeit without notable radiographic or immunogenic responses [90].
NCT01864759 (2022–2023)	I	Adenovirus, single intravenous infusion	Metastatic Uveal Melanoma	**A Phase 1 Trial of Oncolytic Adenovirus ICOVIR-5 Administered Intravenously to Cutaneous and Uveal Melanoma Patients** The systematic administration of ICOVIR-5 resulted in tumor-specific targeting without clinically significant tumor regression [119].
NCT03408587 (2018–2019)	I	Coxsackievirus (CVA21), intravenous infusion	Uveal Melanoma Metastatic to Liver	**CAVATAK^®^ and Ipilimumab in Uveal Melanoma Metastatic to the Liver (VLA-024 CLEVER)** The intravenous administration of CAVATAK and ipilimumab had a manageable safety profile but lacked clinical benefits [120].

**Table 5:** Summary of clinical trials of immunotherapy for the treatment of uveal melanoma and retinoblastoma.

Clinical Trial Identification (Start Year–End Year)	Phase	Treatment	Study Title/ Study Conclusion
Uveal melanoma			
NCT06246149 (2024–2032)	III	Tebentafusp	**Adjuvant Tebentafusp in High-Risk Ocular Melanoma**
NCT06414590 (2024–2028)	II	Tebentafusp	**Neoadjuvant Tebentafusp for Uveal Melanoma**
NCT06070012 (2024–2029)	II	Tebentafusp	Tebentafusp in HLA-A*0201 Positive Previously Untreated Metastatic Uveal Melanoma
NCT05315258 (2022–2026)	II	Tebentafusp	**Tebentafusp in Molecular Relapsed Disease (MRD) Melanoma**
NCT02570308 (2016–2022)	I/II	Tebentafusp	**A Study of the Intra-Patient Escalation Dosing Regimen with IMCgp100 in Patients With Advanced Uveal Melanoma** Tebentafusp has an acceptable safety profile in patients with previously treated UM, and ctDNA data suggest early indications of a clinical benefit [190].
NCT06627244 (2024–2030)	II	Combination of Tebentafusp and Radioembolization	**Study of Tebentafusp and Radioembolization in the Treatment of Metastatic Uveal Melanoma**
NCT06581406 (2025–2031)	II/III	Combination of RP2 and Nivolumab vs. Ipilimumab	**A Randomized, Phase 2/3 Study to Investigate the Efficacy and Safety of RP2 in Combination with Nivolumab in Immune Checkpoint Inhibitor-Naïve Adult Patients With Metastatic Uveal Melanoma**
NCT06519266 (2024–2030)	III	Combination of PHP, Ipilimumab, and Nivolumab vs. Ipilimumab	**PHP in Combination with IPI1/NIVO3 Compared to IPI3/NIVO1 Only in Patients With Uveal Melanoma Liver Metastases**
NCT03611868 (2018–2025)	I/II	APG-115 Monotherapy vs. APG-115 Combined with Pembrolizumab	**A Study of APG-115 in as a Monotherapy or Combination with Pembrolizumab in Patients With Metastatic Melanomas or Advanced Solid Tumors**
NCT05282901 (2022–2028)	II	Combination of Pembrolizumab and Lenvatinib	**Efficacy and Safety of Pembrolizumab in Combination with Lenvatinib in Metastatic Uveal Melanoma Patients (PLUME)**
NCT05308901 (2022–2027)	II	Combination of Pembrolizumab and Lenvatinib	**Lenvatinib Plus Pembrolizumab In Patients With Immune Checkpoint Inhibitor Naïve Metastatic Uveal Melanoma**
NCT05524935 (2022–2026)	II	Combination of Pembrolizumab and Olaparib	**Olaparib in Combination With Pembrolizumab for Advanced Uveal Melanoma**
NCT04336241 (2019–2028)	I	RP2 Monotherapy vs. RP2 Combined with Nivolumab	**Study of RP2 Monotherapy and RP2 in Combination With Nivolumab in Patients With Solid Tumors**
NCT04283890 (2019–2024)	I/II	Combination of Melphalan (PHP), Ipilimumab, and Nivolumab	**PHP and Immunotherapy in Metastasized UM (CHOPIN)**
NCT03472586 (2018–2024)	II	Combination of Ipilimumab, Nivolumab, and Immunoembolization	**Ipilimumab and Nivolumab With Immunoembolization in Treating Participants With Metastatic Uveal Melanoma in the Liver**
NCT04463368 (2021–2024)	I	Combination of Melphalan (PHP), Ipilimumab, and Nivolumab	**Isolated Hepatic Perfusion in Combination with Ipilimumab and Nivolumab in Patients with Uveal Melanoma Metastases**
NCT04552223 (2020–2026)	II	Combination of Nivolumab and Relatlimab	**Nivolumab Plus Relatlimab in Patients with Metastatic Uveal Melanoma**
NCT02697630 (2018–2023)	II	Combination of Pembrolizumab and Entinostat	**Efficacy Study of Pembrolizumab with Entinostat to Treat Metastatic Melanoma of the Eye** A combination treatment of pembrolizumab and entinostat in patients with metastatic uveal melanoma had manageable toxicities with a subset of patients (*BAP1+* tumors) showing an enhanced response and prolonged survival [191].
NCT03922880 (2019–2023)	I	Combination of ADI PEG20, Ipilimumab, and Nivolumab	**Study of Immunotherapy Plus ADI-PEG 20 for the Treatment of Advanced Uveal Melanoma** A combination treatment of ADI-PEG20 in combination with ipiliumumab and nivolumab had a tolerable safety profile but did not lead to any noticeable clinical benefit [192].
NCT02626962 (2016–2021)	II	Combination of Ipilimumab and Nivolumab	**Trial of Nivolumab in Combination with Ipilimumab in Subjects with Previously Untreated Metastatic Uveal Melanoma** Nivolumab combined with ipilimumab used in a first-line therapy for metastatic UM resulted in a moderate improvement in the overall survival in comparison to standard chemotherapy [193].
NCT01034787 (2009–2017)	II	Tremelimumab (anti-CTLA-4)	**Study of AntiCTLA4 in Patients with Unresectable or Metastatic Uveal Melanoma** There was a lack of progression-free survival and overall response in patients with unresectable metastatic UM receiving Tremelimumab [194].
NCT02158520 (2013–2019)	II	Combination of Nab-paclitaxel and Bevacizumab vs. Ipilimumab	**Nab-Paclitaxel and Bevacizumab or Ipilimumab as First-Line Therapy in Treating Patients with Stage IV Melanoma That Cannot Be Removed by Surgery** The first-line treatment of Nab-paclitaxel and bevacizumab compared to ipilimumab resulted in no significant differences in the overall survival and progression-free survival.
NCT02519322 (2016–2023)	II	Combination of Nivolumab and Relatlimib vs. Nivolumab and Ipilimumab	**Neoadjuvant and Adjuvant Checkpoint Blockade** Ipilimumab followed by adjuvant nivolumab or relatlimab had a 70% pathological response rate with a tolerable safety profile in patients with late-stage resectable melanomas [195].
NCT01585194 (2012–2024)	II	Combination of Nivolumab and Ipilimumab	**Nivolumab and Ipilimumab in Treating Patients with Metastatic Uveal Melanoma** Nivolumab and ipilimumab combination therapy resulted in clinically significant anti-tumor activity in patients with metastatic UM [196].
NCT03070392 (2017–2025)	II	Tebentafusp vs. Decarbazine, Ipilimumab, or Pembrolizumab	**Safety and Efficacy of IMCgp100 Versus Investigator Choice in Advanced Uveal Melanoma** Treatment with tebentafusp resulted in an increased overall survival but similar progression-free survival compared to controls receiving dacarbazine, ipilimumab, or pembrolizumab [197].
NCT03611868 (2024–2030)	I/II	Combination of Cemiplimab (anti-PD-1) and Ziv-Aflibercept	**Study of Cemiplimab Plus Ziv-Aflibercept for Subjects With Metastatic Uveal Melanoma**
NCT04812470 (2023–2030)	I	TILs	**Hepatic Arterial Infusion of Autologous Tumor Infiltrating Lymphocytes in Patients with Melanoma and Liver Metastases**
NCT05628883 (2022–2026)	I	Combination of TILs, Cyclophosphamide, Fludarabine, and Interleukin-2	**Proof of Concept of Tbio-4101, Lymphodepleting Chemo, IL-2 for Relapsed/Refractory Melanoma**
NCT05576077 (2023–2025)	I	Combination of TILs and Pembrolizumab	**A Study of Tbio-4101 (TIL) and Pembrolizumab in Patients with Advanced Solid Tumors**
NCT04729543 (2020–2027)	I/II	ACT: MC2 TCR T Cells	**MAGE-C2 TCR T Cell Trial to Treat Melanoma and Head and Neck Cancer**
NCT05607095 (2022–2025)	I	TILs	**Pilot Trial of Autologous Tumor Infiltrating Lymphocytes (LN-144) for Patients with Metastatic Uveal Melanoma**
NCT03467516 (2018–2027)	II	TILs	**Adoptive Transfer of Tumor Infiltrating Lymphocytes for Metastatic Uveal Melanoma**
NCT05607095 (2022–2025)	I	TILs	**A Study of LN-144 or LN-145 in People With Advanced Uveal Melanoma, Undifferentiated Pleomorphic Sarcoma, or Dedifferentiated Liposarcoma**
NCT03068624 (2017–2025)	I	Combination of CD8+ SLC45A2-Specific TILs, Ipilimumab, Aldesleukin, and Cyclophosphamide	**Autologous CD8+ SLC45A2-Specific T Lymphocytes with Cyclophosphamide, Aldesleukin, and Ipilimumab in Treating Patients With Metastatic Uveal Melanoma**
NCT05903937 (2023–2029)	I	TILs	**Locoregional Administration of TIL and Lymphodepletion in Patients With Melanoma and Liver Metastases (HAITILS-PHP)**
NCT04119024 (2019–2025)	I	IL13Ralpha2 CAR T Cells	**Gene Modified Immune Cells (IL13Ralpha2 CAR T Cells) After Conditioning Regimen for the Treatment of Stage IIIC or IV Melanoma or Metastatic Solid Tumors**
NCT03635632 (2019–2038)	I	C7R-GD2.CAR T Cells	**C7R-GD2.CART Cells for Patients with Relapsed or Refractory Neuroblastoma and Other GD2 Positive Cancers (GAIL-N)**
Retinoblastoma			
NCT04483778 (2020–2040)	I	Combination of B7H3 CAR T Cells and Pembrolizumab	**B7H3 CAR T Cell Immunotherapy for Recurrent/Refractory Solid Tumors in Children and Young Adults**
NCT03618381 (2019–2040)	I	EGFR806 CAR T Cells	**EGFR806 CAR T Cell Immunotherapy for Recurrent/Refractory Solid Tumors in Children and Young Adults**

**Table 6:** Summary of clinical trials of targeted immunotherapy for the treatment of uveal melanoma.

Clinical Trial Identification	Phase	Treatment	Study Title
Protein kinase C inhibitors			
NCT05987332	II/III	Neoadjuvant/adjuvant darovasertib	IDE196 (Darovasertib) in Combination With Crizotinib as First-line Therapy in Metastatic Uveal Melanoma
NCT05907954	II	Neoadjuvant/adjuvant darovasertib	(Neo)Adjuvant IDE196 (Darovasertib) in Patients With Localized Ocular Melanoma
NCT03947385	I/II	Neoadjuvant/adjuvant darovasertib	Study of IDE196 in Patients with Solid Tumors Harboring GNAQ/11 Mutations or PRKC Fusions
NCT01430416	I	Sotrastaurin	A Phase 1 Study of AEB071, an Oral Protein Kinase C Inhibitor, in Patients With Metastatic Uveal Melanoma
MEK inhibitors			
NCT02768766	Ib	Selumetinib	Multi-Center Phase Ib Study of Intermittent Dosing of the MEK Inhibitor, Selumetinib, in Patients With Advanced Uveal Melanoma Not Previously Treated With a MEK Inhibitor
NCT01328106	II	Trametinib	A Single-Arm, Open-Label, Multi-Center Study to Investigate the Objective Response Rate, Safety, and Pharmacokinetics of GSK1120212, a MEK Inhibitor, in Subjects With Metastatic UvealMelanoma or With Mutation-Positive GNAQ or GNA11 Metastatic Melanoma
NCT01143402	II	Selumetinib	Randomized Phase II Trial of Temozolomide Versus Hyd-Sulfate AZD6244 [NSC 748727] in Patients With Metastatic Uveal Melanoma
ERK inhibitors			
NCT03417739	II	Ulixertinib	A Phase II Study of BVD-523 in Metastatic Uveal Melanoma
Combination therapies			
NCT01801358	Ib/II	Sotrastaurin and binimetinib combination therapy	A Phase Ib/II, Open-label, Multicenter Study of AEB071 and MEK162 in Adult Patients With Metastatic Uveal Melanoma
NCT02273219	Ib	Sotrastaurin and alpesilib combination therapy	Phase Ib Trial of AEB071, a PKC Inhibitor, in Combination With BYL719, a PI3Kα Inhibitor, in Patients With Metastatic Uveal Melanoma
NCT04720417	II	Defactinib and VS-6766 combination therapy	A Phase II Trial of Defactinib (VS-6063) Combined With VS-6766 (CH5126766) in Patients With Metastatic Uveal Melanoma
NCT05677373	I/II	PLX2853 and Trametinib combination therapy	Phase I/II Study of BET and MEK Inhibition in Advanced UvealMelanoma
NCT01979523	II	Trametinib and uprosertib combination therapy	A Randomized Two-Arm Phase II Study of Trametinib Alone and in Combination With GSK2141795 in Patients With Advanced UvealMelanoma

## References

[1] Bornfeld N., Biewald E., Bauer S., Temming P., Lohmann D., Zeschnigk M. (2018). The Interdisciplinary Diagnosis and Treatment of Intraocular Tumors. Dtsch. Arztebl. Int..

[2] Davies L., Gray D., Spiller D., White M.R., Damato B., Grierson I., Paraoan L. (2009). P53 apoptosis mediator PERP: Localization, function and caspase activation in uveal melanoma. J. Cell. Mol. Med..

[3] Zhang S., Wang K., Zhu X., Cherepanoff S., Conway R.M., Madigan M.C., Zhu L., Murray M., Zhou F. (2022). The unfolded protein response and the biology of uveal melanoma. Biochimie.

[4] Goesmann L., Refaian N., Bosch J.J., Heindl L.M. (2023). Characterization and Quantitation of the Tumor Microenvironment of Uveal Melanoma. Biology.

[5] Tang S., Zhang Y., Huang S., Zhu T., Huang X. (2024). Single cell RNA-sequencing in uveal melanoma: Advances in heterogeneity, tumor microenvironment and immunotherapy. Front. Immunol..

[6] Kaewkhaw R., Rojanaporn D. (2020). Retinoblastoma: Etiology, modeling, and treatment. Cancers.

[7] Tort F., Bartkova J., Sehested M., Ørntoft T., Lukas J., Bartek J. (2006). Retinoblastoma pathway defects show differential ability to activate the constitutive DNA damage response in human tumorigenesis. Cancer Res..

[8] Mao P., Shen Y., Xu X., Zhong J. (2022). Comprehensive analysis of the immune cell infiltration landscape and immune-related methylation in retinoblastoma. Front. Genet..

[9] Cruz-Gálvez C.C., Ordaz-Favila J.C., Villar-Calvo V.M., Cancino-Marentes M.E., Bosch-Canto V. (2022). Retinoblastoma: Review and new insights. Front. Oncol..

[10] Wu C., Yang J., Xiao W., Jiang Z., Chen S., Guo D., Zhang P., Liu C., Yang H., Xie Z. (2022). Single-cell characterization of malignant phenotypes and microenvironment alteration in retinoblastoma. Cell Death Dis..

[11] Silva-Rodríguez P., Fernández-Díaz D., Bande M., Pardo M., Loidi L., Blanco-Teijeiro M.J. (2022). GNAQ and GNA11 Genes: A Comprehensive Review on Oncogenesis, Prognosis and Therapeutic Opportunities in Uveal Melanoma. Cancers.

[12] Marković L., Bukovac A., Varošanec A.M., Šlaus N., Pećina-Šlaus N. (2023). Genetics in ophthalmology: Molecular blueprints of retinoblastoma. Human. Genom..

[13] Zhou M., Tang J., Fan J., Wen X., Shen J., Jia R., Chai P., Fan X. (2024). Recent progress in retinoblastoma: Pathogenesis, presentation, diagnosis and management. Asia-Pac. J. Ophthalmol..

[14] Byroju V.V., Nadukkandy A.S., Cordani M., Kumar L.D. (2023). Retinoblastoma: Present scenario and future challenges. Cell Commun. Signal..

[15] Kulbay M., Marcotte E., Remtulla R., Lau T.H.A., Paez-Escamilla M., Wu K.Y., Burnier M.N. (2024). Uveal Melanoma: Comprehensive Review of Its Pathophysiology, Diagnosis, Treatment, and Future Perspectives. Biomedicines.

[16] Yang J., Manson D.K., Marr B.P., Carvajal R.D. (2018). Treatment of uveal melanoma: Where are we now?. Ther. Adv. Med. Oncol..

[17] Soliman N., Mamdouh D., Elkordi A. (2023). Choroidal Melanoma: A Mini Review. Medicines.

[18] Zemba M., Dumitrescu O.-M., Gheorghe A.G., Radu M., Ionescu M.A., Vatafu A., Dinu V. (2023). Ocular Complications of Radiotherapy in Uveal Melanoma. Cancers.

[19] Tarlan B., Kiratli H. (2012). Current treatment of choroidal melanoma. Expert. Rev. Ophthalmol..

[20] Caminal J.M., Lorenzo D., Gutierrez C., Slocker A., Piulats J.M., Cobos E., Garcia-Bru P., Morwani R., Santamaria J.F., Arias L. (2022). Local Resection in Choroidal Melanoma: A Review. JCM.

[21] Damato B. (2018). Ocular treatment of choroidal melanoma in relation to the prevention of metastatic death—A personal view. Prog. Retin. Eye Res..

[22] Vidoris A.A.C., Maia A., Lowen M., Morales M., Isenberg J., Fernandes B.F., Belfort R.N. (2017). Outcomes of primary endoresection for choroidal melanoma. Int. J. Retin. Vitr..

[23] Karkhaneh R., Chams H., Amoli F.A., Riazi-Esfahani M., Ahmadabadi M.N., Mansouri M.R., Nouri K., Karkhaneh A. (2007). Long-term Surgical Outcome Of Posterior Choroidal Melanoma Treated By Endoresection. Retina.

[24] Baum S.H., Westekemper H., Bechrakis N.E., Mohr C. (2022). Conjunctival and uveal melanoma: Survival and risk factors following orbital exenteration. Eur. J. Ophthalmol..

[25] Shields C.L., Shields J.A., Albert D.M., Miller J.W., Azar D.T., Young L.H. (2022). Enucleation for Uveal Melanoma. Albert and Jakobiec’s Principles and Practice of Ophthalmology.

[26] Foti P.V., Travali M., Farina R., Palmucci S., Spatola C., Liardo R.L.E., Milazzotto R., Raffaele L., Salamone V., Caltabiano R. (2021). Diagnostic methods and therapeutic options of uveal melanoma with emphasis on MR imaging—Part II: Treatment indications and complications. Insights Imaging.

[27] Maheshwari A., Finger P.T. (2023). Laser treatment for choroidal melanoma: Current concepts. Surv. Ophthalmol..

[28] Bilmin K., Synoradzki K.J., Czarnecka A.M., Spałek M.J., Kujawska T., Solnik M., Merks P., Toro M.D., Rejdak R., Fiedorowicz M. (2021). New Perspectives for Eye-Sparing Treatment Strategies in Primary Uveal Melanoma. Cancers.

[29] Roelofs K.A., Fabian I.D., Arora A.K., Cohen V.M.L., Sagoo M.S. (2021). Long-term Outcomes of Small Pigmented Choroidal Melanoma Treated with Primary Photodynamic Therapy. Ophthalmol. Retin..

[30] Yordi S., Soto H., Bowen R.C., Singh A.D. (2021). Photodynamic therapy for choroidal melanoma: What is the response rate?. Surv. Ophthalmol..

[31] Blasi M., Pagliara M., Lanza A., Sammarco M., Caputo C., Grimaldi G., Scupola A. (2018). Photodynamic Therapy in Ocular Oncology. Biomedicines.

[32] Shields C.L., Lally S.E., Leahey A.M., Jabbour P.M., Caywood E.H., Schwendeman R., Shields J.A. (2014). Targeted retinoblastoma management: When to use intravenous, intra-arterial, periocular, and intravitreal chemotherapy. Curr. Opin. Ophthalmol..

[33] Ebrahimi K.B., Hang A., O’Brien J.M., Albert D.M., Miller J.W., Azar D.T., Young L.H. (2022). Current Management of Retinoblastoma. Albert and Jakobiec’s Principles and Practice of Ophthalmology.

[34] Wei R., Li M., Yang W., Xu H., Choi J., Zhou X. (2021). Case Report: Phototherapeutic Keratectomy for Band Keratopathy Secondary to Chemo-Laser-Cryotherapy for Retinoblastoma. Front. Med..

[35] Shields C.L., Shields J.A. (2010). Retinoblastoma management: Advances in enucleation, intravenous chemoreduction, and intra-arterial chemotherapy. Curr. Opin. Ophthalmol..

[36] Kritfuangfoo T., Rojanaporn D. (2024). Update on chemotherapy modalities for retinoblastoma: Progress and challenges. Asia-Pac. J. Ophthalmol..

[37] Manukonda R., Narayana R.V., Kaliki S., Mishra D.K., Vemuganti G.K. (2022). Emerging therapeutic targets for retinoblastoma. Expert Opin. Ther. Targets.

[38] Zhao J., Feng Z., Leung G., Gallie B.L. (2021). Retinoblastoma Survival Following Primary Enucleation by AJCC Staging. Cancers.

[39] Banerjee S.C., Pottenger E., Petriccione M., Chou J.F., Ford J.S., Sklar C.A., Robison L.L., Kleinerman R.A., Oeffinger K.C., Francis J.H. (2020). Impact of enucleation on adult retinoblastoma survivors’ quality of life: A qualitative study of survivors’ perspectives. Palliat. Support. Care.

[40] Appukuttan B., Biswas J., Khetan V. (2013). Enucleation in retinoblastoma: Pros and cons. Expert Rev. Ophthalmol..

[41] Gunalp I., Gündüz K., Dürük K. (1996). Orbital exenteration: A review of 429 cases. Int. Ophthalmol..

[42] Kaliki S., Palkonda V.A.R. (2018). Management of retinoblastoma with extraocular tumour extension. Community Eye Health.

[43] Bhakuni Y.S., Sharma K.C., Rajappa S.K., Ram D., Dewan A.K., Chand R., Maheshwari U., Jajodia A., Babu Koyyala V.P. (2021). Total Orbital Exenteration—Experience from a Tertiary Cancer Care Center in Northern India. Oncol. J. India.

[44] Manjandavida F., Stathopoulos C., Zhang J., Honavar S., Shields C. (2019). Intra-arterial chemotherapy in retinoblastoma—A paradigm change. Indian J. Ophthalmol..

[45] Liang T., Zhang X., Li J., Hua X., Zhao P., Ji X. (2022). Intra-Arterial Chemotherapy as Primary Treatment for Advanced Unilateral Retinoblastoma in China. Front. Med..

[46] Daniels A.B., Froehler M.T., Kaczmarek J.V., Bogan C.M., Santapuram P.R., Pierce J.M., Chen S.-C., Schremp E.A., Boyd K.L., Tao Y.K. (2021). Efficacy, Toxicity, and Pharmacokinetics of Intra-Arterial Chemotherapy Versus Intravenous Chemotherapy for Retinoblastoma in Animal Models and Patients. Trans. Vis. Sci. Tech..

[47] Ancona-Lezama D., Dalvin L., Shields C. (2020). Modern treatment of retinoblastoma: A 2020 review. Indian J. Ophthalmol..

[48] Tanveer S., Zafar F., Bibi H., Haroon H., Ahmad O., Iqbal M.S., Zakir Z., Khilji M., Tanveer S., Hassan R.E. (2024). Advancements in Retinoblastoma Treatment: Unraveling the Potential of Intravitreal Chemotherapy. Cureus.

[49] Yousef Y.A., Halliday W., Chan H.S.L., Héon E., Gallie B.L., Dimaras H. (2013). No ocular motility complications after subtenon topotecan with fibrin sealant for retinoblastoma. Can. J. Ophthalmol..

[50] Munier F.L., Gaillard M.-C., Decembrini S., Bongiovanni M., Beck-Popovic M. (2017). Intracameral Chemotherapy (Melphalan) for Aqueous Seeding in Retinoblastoma: Bicameral Injection Technique and Related Toxicity in a Pilot Case Study. Ocul. Oncol. Pathol..

[51] Stathopoulos C., Beck-Popovic M., Moulin A.P., Munier F.L. (2024). Ten-year experience with intracameral chemotherapy for aqueous seeding in retinoblastoma: Long-term efficacy, safety and toxicity. Br. J. Ophthalmol..

[52] Cieślik K., Rogowska A., Danowska M., Trocka K., Rutynowska O., Dembowska-Bagińska B., Kołodziejczyk-Gietka A., Charzyńska I., Hautz W. (2022). Episcleral brachytherapy for intraocular retinoblastoma with 106Ruthenium plaque: Analysis of 13 procedures. Klin. Ocz..

[53] Simpson E.R., Gallie B., Laperrierre N., Beiki-Ardakani A., Kivelä T., Raivio V., Heikkonen J., Desjardins L., Dendale R., Mazal A. (2014). The American Brachytherapy Society consensus guidelines for plaque brachytherapy of uveal melanoma and retinoblastoma. Brachytherapy.

[54] Kim J.W., Murphree A.L., Singh A.D., Singh A.D., Murphree A.L., Damato B.E. (2015). Retinoblastoma: Treatment Options. Clinical Ophthalmic Oncology.

[55] Kim J.-Y., Park Y. (2015). Treatment of Retinoblastoma: The Role of External Beam Radiotherapy. Yonsei Med. J..

[56] Shields C.L., Shields J.A., Kiratli H., De Potter P.V. (1995). Treatment of Retinoblastoma With Indirect Ophthalmoscope Laser Photocoagulation. J. Pediatr. Ophthalmol. Strabismus.

[57] Kaneko A. (2003). Eye-Preservation Treatment of Retinoblastoma with Vitreous Seeding. Jpn. J. Clin. Oncol..

[58] Choi S.Y., Kim M.-S., Yoo S., Cho C., Ji Y., Kim K., Seo Y., Park K.D., Lee J., Lee T.-W. (2010). Long Term Follow-up Results of External Beam Radiotherapy as Primary Treatment for Retinoblastoma. J. Korean Med. Sci..

[59] Journee-de Korver J.G., Oosterhuis J.A., De Wolff-Rouendaal D., Kemme H. (1997). Histopathological findings in human choroidal melanomas after transpupillary thermotherapy. Br. J. Ophthalmol..

[60] Abramson D.H., Schefler A.C. (2004). Transpupillary thermotherapy as initial treatment for small intraocular retinoblastoma. Ophthalmology.

[61] Shields C.L. (1999). Thermotherapy for Retinoblastoma. Arch. Ophthalmol..

[62] Hasanreisoglu M., Saktanasate J., Schwendeman R., Shields J.A., Shields C.L. (2015). Indocyanine Green-Enhanced Transpupillary Thermotherapy for Retinoblastoma: Analysis of 42 Tumors. J. Pediatr. Ophthalmol. Strabismus.

[63] Shields J.A., Parsons H., Shields C.L., Giblin M.E. (1989). The Role of Cryotherapy in the Management of Retinoblastoma. Am. J. Ophthalmol..

[64] Shields J.A. (1990). The Role of Photocoagulation in the Management of Retinoblastoma. Arch. Ophthalmol..

[65] Soliman S., Feng Z.X., Gallie B.L. (2020). Evaluating laser photocoagulation for discrete retinoblastoma tumors. Investig. Ophthalmol. Vis. Sci..

[66] Zhang M.G., Kuznetsoff J.N., Owens D.A., Gallo R.A., Kalahasty K., Cruz A.M., Kurtenbach S., Correa Z.M., Pelaez D., Harbour J.W. (2022). Early Mechanisms of Chemoresistance in Retinoblastoma. Cancers.

[67] Fu Y., Xiao W., Mao Y. (2022). Recent Advances and Challenges in Uveal Melanoma Immunotherapy. Cancers.

[68] Schefler A.C., Kim R.S. (2021). Recent advancements in the management of retinoblastoma and uveal melanoma. Fac. Rev..

[69] Wang X., Shen Y., Wan X., Hu X., Cai W.-Q., Wu Z., Xin Q., Liu X., Gui J., Xin H.-Y. (2023). Oncolytic virotherapy evolved into the fourth generation as tumor immunotherapy. J. Transl. Med..

[70] Lin D., Shen Y., Liang T. (2023). Oncolytic virotherapy: Basic principles, recent advances and future directions. Sig Transduct. Target. Ther..

[71] Zhu X., Fan C., Xiong Z., Chen M., Li Z., Tao T., Liu X. (2023). Development and application of oncolytic viruses as the nemesis of tumor cells. Front. Microbiol..

[72] Pascual-Pasto G., Bazan-Peregrino M., Olaciregui N.G., Restrepo-Perdomo C.A., Mato-Berciano A., Ottaviani D., Weber K., Correa G., Paco S., Vila-Ubach M. (2019). Therapeutic targeting of the RB1 pathway in retinoblastoma with the oncolytic adenovirus VCN-01. Sci. Transl. Med..

[73] Seyed-Khorrami S.-M., Azadi A., Rastegarvand N., Habibian A., Soleimanjahi H., Łos M.J. (2023). A promising future in cancer immunotherapy: Oncolytic viruses. Eur. J. Pharmacol..

[74] Yan Z., Zhang Z., Chen Y., Xu J., Wang J., Wang Z. (2024). Enhancing cancer therapy: The integration of oncolytic virus therapy with diverse treatments. Cancer Cell Int..

[75] Hwang J.K., Hong J., Yun C.-O. (2020). Oncolytic Viruses and Immune Checkpoint Inhibitors: Preclinical Developments to Clinical Trials. Int. J. Mol. Sci..

[76] Howells A., Marelli G., Lemoine N.R., Wang Y. (2017). Oncolytic Viruses—Interaction of Virus and Tumor Cells in the Battle to Eliminate Cancer. Front. Oncol..

[77] Hemminki O., Dos Santos J.M., Hemminki A. (2020). Oncolytic viruses for cancer immunotherapy. J. Hematol. Oncol..

[78] Kaufman H.L., Kohlhapp F.J., Zloza A. (2015). Oncolytic viruses: A new class of immunotherapy drugs. Nat. Rev. Drug Discov..

[79] Tian Y., Xie D., Yang L. (2022). Engineering strategies to enhance oncolytic viruses in cancer immunotherapy. Sig Transduct. Target. Ther..

[80] Chu R.L., Post D.E., Khuri F.R., Van Meir E.G. (2004). Use of Replicating Oncolytic Adenoviruses in Combination Therapy for Cancer. Clin. Cancer Res..

[81] Scanlan H., Coffman Z., Bettencourt J., Shipley T., Bramblett D.E. (2022). Herpes simplex virus 1 as an oncolytic viral therapy for refractory cancers. Front. Oncol..

[82] Nemerow G.R., Pache L., Reddy V., Stewart P.L. (2009). Insights into adenovirus host cell interactions from structural studies. Virology.

[83] Mozzi A., Cagliani R., Pontremoli C., Forni D., Saulle I., Saresella M., Pozzoli U., Cappelletti G., Vantaggiato C., Clerici M. (2022). Simplexviruses Successfully Adapt to Their Host by Fine-Tuning Immune Responses. Mol. Biol. Evol..

[84] Jiao X., Sui H., Lyons C., Tran B., Sherman B.T., Imamichi T. (2019). Complete Genome Sequence of Herpes Simplex Virus 1 Strain McKrae. Microbiol. Resour. Announc..

[85] Kennedy M.A., Parks R.J. (2009). Adenovirus Virion Stability and the Viral Genome: Size Matters. Mol. Ther..

[86] Antar A.A.R., Konopka J.L., Campbell J.A., Henry R.A., Perdigoto A.L., Carter B.D., Pozzi A., Abel T.W., Dermody T.S. (2009). Junctional Adhesion Molecule-A Is Required for Hematogenous Dissemination of Reovirus. Cell Host Microbe.

[87] McSherry E.A., McGee S.F., Jirstrom K., Doyle E.M., Brennan D.J., Landberg G., Dervan P.A., Hopkins A.M., Gallagher W.M. (2009). JAM-A expression positively correlates with poor prognosis in breast cancer patients. Int. J. Cancer.

[88] Luo D., Wang H., Wang Q., Liang W., Liu B., Xue D., Yang Y., Ma B. (2022). Senecavirus A as an Oncolytic Virus: Prospects, Challenges and Development Directions. Front. Oncol..

[89] Zeng W., Yan Q., Du P., Yuan Z., Sun Y., Liu X., Zhang L., Liu X., Ding H., Yi L. (2023). Evolutionary dynamics and adaptive analysis of Seneca Valley virus. Infect. Genet. Evol..

[90] Smith K.E.R., Peng K.-W., Pulido J.S., Weisbrod A.J., Strand C.A., Allred J.B., Newsom A.N., Zhang L., Packiriswamy N., Kottke T. (2023). A phase I oncolytic virus trial with vesicular stomatitis virus expressing human interferon beta and tyrosinase related protein 1 administered intratumorally and intravenously in uveal melanoma: Safety, efficacy, and T cell responses. Front. Immunol..

[91] Zhang Y., Nagalo B.M. (2022). Immunovirotherapy Based on Recombinant Vesicular Stomatitis Virus: Where Are We?. Front. Immunol..

[92] Bertram M.R., Rodgers C., Reed K., Velazquez-Salinas L., Pelzel-McCluskey A., Mayo C., Rodriguez L. (2023). Vesicular stomatitis Indiana virus near-full-length genome sequences reveal low genetic diversity during the 2019 outbreak in Colorado, USA. Front. Vet. Sci..

[93] Mathis J.M., Stoff-Khalili M.A., Curiel D.T. (2005). Oncolytic adenoviruses—Selective retargeting to tumor cells. Oncogene.

[94] Haisma H.J., Grill J., Curiel D.T., Hoogeland S., Van Beusechem V.W., Pinedo H.M., Gerritsen W.R. (2000). Targeting of adenoviral vectors through a bispecific single-chain antibody. Cancer Gene Ther..

[95] Nakano K., Asano R., Tsumoto K., Kwon H., Goins W.F., Kumagai I., Cohen J.B., Glorioso J.C. (2005). Herpes Simplex Virus Targeting to the EGF Receptor by a gD-Specific Soluble Bridging Molecule. Mol. Ther..

[96] Yang M., Yang C.S., Guo W., Tang J., Huang Q., Feng S., Jiang A., Xu X., Jiang G., Liu Y.Q. (2017). A novel fiber chimeric conditionally replicative adenovirus-Ad5/F35 for tumor therapy. Cancer Biol. Ther..

[97] Cheng P.-H., Wechman S., McMasters K., Zhou H. (2015). Oncolytic Replication of E1b-Deleted Adenoviruses. Viruses.

[98] Garber K. (2006). China Approves World’s First Oncolytic Virus Therapy For Cancer Treatment. J. Natl. Cancer Inst..

[99] Dave R.V., Jebar A.H.S., Jennings V.A., Adair R.A., West E.J., Errington-Mais F., Toogood G.J., Melcher A.A. (2014). Viral warfare! Front-line defence and arming the immune system against cancer using oncolytic vaccinia and other viruses. Surgeon.

[100] Guo Z.S., Liu Z., Bartlett D.L. (2014). Oncolytic Immunotherapy: Dying the Right Way is a Key to Eliciting Potent Antitumor Immunity. Front. Oncol..

[101] Hong I.-S. (2016). Stimulatory versus suppressive effects of GM-CSF on tumor progression in multiple cancer types. Exp. Mol. Med..

[102] Pol J., Kroemer G., Galluzzi L. (2016). First oncolytic virus approved for melanoma immunotherapy. OncoImmunology.

[103] Ferrucci P.F., Pala L., Conforti F., Cocorocchio E. (2021). Talimogene Laherparepvec (T-VEC): An Intralesional Cancer Immunotherapy for Advanced Melanoma. Cancers.

[104] Liu Z., Ge Y., Wang H., Ma C., Feist M., Ju S., Guo Z.S., Bartlett D.L. (2018). Modifying the cancer-immune set point using vaccinia virus expressing re-designed interleukin-2. Nat. Commun..

[105] Nguyen H.-M., Guz-Montgomery K., Saha D. (2020). Oncolytic Virus Encoding a Master Pro-Inflammatory Cytokine Interleukin 12 in Cancer Immunotherapy. Cells.

[106] Backhaus P.S., Veinalde R., Hartmann L., Dunder J.E., Jeworowski L.M., Albert J., Hoyler B., Poth T., Jäger D., Ungerechts G. (2019). Immunological Effects and Viral Gene Expression Determine the Efficacy of Oncolytic Measles Vaccines Encoding IL-12 or IL-15 Agonists. Viruses.

[107] Dunn G.P., Koebel C.M., Schreiber R.D. (2006). Interferons, immunity and cancer immunoediting. Nat. Rev. Immunol..

[108] Bourgeois-Daigneault M.-C., Roy D.G., Falls T., Twumasi-Boateng K., St-Germain L.E., Marguerie M., Garcia V., Selman M., Jennings V.A., Pettigrew J. (2016). Oncolytic vesicular stomatitis virus expressing interferon-σ has enhanced therapeutic activity. Mol. Ther.-Oncolytics.

[109] LaRocca C.J., Han J., Gavrikova T., Armstrong L., Oliveira A.R., Shanley R., Vickers S.M., Yamamoto M., Davydova J. (2015). Oncolytic adenovirus expressing interferon alpha in a syngeneic Syrian hamster model for the treatment of pancreatic cancer. Surgery.

[110] Li F., Sheng Y., Hou W., Sampath P., Byrd D., Thorne S., Zhang Y. (2020). CCL5-armed oncolytic virus augments CCR5-engineered NK cell infiltration and antitumor efficiency. J. Immunother. Cancer.

[111] Liu Z., Ravindranathan R., Li J., Kalinski P., Guo Z.S., Bartlett D.L. (2016). CXCL11-Armed oncolytic poxvirus elicits potent antitumor immunity and shows enhanced therapeutic efficacy. OncoImmunology.

[112] O’Neill R.E., Cao X. (2019). Co-stimulatory and co-inhibitory pathways in cancer immunotherapy. Advances in Cancer Research.

[113] Lovatt C., Parker A.L. (2023). Oncolytic Viruses and Immune Checkpoint Inhibitors: The “Hot” New Power Couple. Cancers.

[114] Guo Z.S., Lotze M.T., Zhu Z., Storkus W.J., Song X.-T. (2020). Bi- and Tri-Specific T Cell Engager-Armed Oncolytic Viruses: Next-Generation Cancer Immunotherapy. Biomedicines.

[115] Wang Q., Ma X., Wu H., Zhao C., Chen J., Li R., Yan S., Li Y., Zhang Q., Song K. (2022). Oncolytic adenovirus with MUC16-BiTE shows enhanced antitumor immune response by reversing the tumor microenvironment in PDX model of ovarian cancer. OncoImmunology.

[116] Russell S.J., Barber G.N. (2018). Oncolytic Viruses as Antigen-Agnostic Cancer Vaccines. Cancer Cell.

[117] Liu W., Dai E., Liu Z., Ma C., Guo Z.S., Bartlett D.L. (2020). In Situ Therapeutic Cancer Vaccination with an Oncolytic Virus Expressing Membrane-Tethered IL-2. Mol. Ther.-Oncolytics.

[118] (2024). Theriva^TM^ Biologics Receives Rare Pediatric Drug Designation by the U.S. FDA for VCN-01 for the Treatment of Retinoblastoma.

[119] García M., Moreno R., Gil-Martin M., Cascallò M., De Olza M.O., Cuadra C., Piulats J.M., Navarro V., Domenech M., Alemany R. (2019). A Phase 1 Trial of Oncolytic Adenovirus ICOVIR-5 Administered Intravenously to Cutaneous and Uveal Melanoma Patients. Hum. Gene Ther..

[120] Lutzky J., Sullivan R.J., Cohen J.V., Ren Y., Li A., Haq R. (2023). Phase 1b study of intravenous coxsackievirus A21 (V937) and ipilimumab for patients with metastatic uveal melanoma. J. Cancer Res. Clin. Oncol..

[121] Liu S., Li M., Sun F., Zhang J., Liu F. (2023). Enhancing the immune effect of oHSV-1 therapy through TLR3 signaling in uveal melanoma. J. Cancer Res. Clin. Oncol..

[122] Liu S., Liu F., Zhao M., Zhang J. (2020). Antitumor Efficacy of Oncolytic Herpes Virus Type 1 Armed with GM-CSF in Murine Uveal Melanoma Xenografts. Cancer Manag. Res..

[123] Liu S., Zhang J., Fang S., Zhang Q., Zhu G., Tian Y., Zhao M., Liu F. (2021). Macrophage polarization contributes to the efficacy of an oncolytic HSV-1 targeting human uveal melanoma in a murine xenograft model. Exp. Eye Res..

[124] Liu S., Zhang J., Fang S., Su X., Zhang Q., Zhu G., Zhu L., Zhao M., Liu F. (2020). Antitumor efficacy of oncolytic HSV-1 expressing cytosine deaminase is synergistically enhanced by DPD down-regulation and EMT inhibition in uveal melanoma xenograft. Cancer Lett..

[125] Cullinan A., Lindstrom M., Sabet S., Albert D., Brandt C. (2004). Evaluation of the antitumor effects of Herpes simplex virus lacking ribonucleotide reductase in a murine retinoblastoma model. Curr. Eye Res..

[126] Ji X., Zhang J., Cheng L., Wei F., Li H., Liu X., Chen X., Li C., Wang Y., Huang Q. (2009). Oncolytic adenovirus delivering herpes simplex virus thymidine kinase suicide gene reduces the growth of human retinoblastoma in an in vivo mouse model. Exp. Eye Res..

[127] Cascallo M., Alonso M.M., Rojas J.J., Perez-Gimenez A., Fueyo J., Alemany R. (2007). Systemic Toxicity–Efficacy Profile of ICOVIR-5, a Potent and Selective Oncolytic Adenovirus Based on the pRB Pathway. Mol. Ther..

[128] Cun B., Song X., Jia R., Zhao X., Wang H., Ge S., Fan X. (2012). Combination of oncolytic adenovirus and dacarbazine enhances antitumor ability against uveal melanoma cells via cell cycle block. Cancer Biol. Ther..

[129] Li Y., He J., Qiu C., Shang Q., Qian G., Fan X., Ge S., Jia R. (2019). The oncolytic virus H101 combined with *GNAQ* siRNA-mediated knockdown reduces uveal melanoma cell viability. J. Cell. Biochem..

[130] Ildefonso C.J., Kong L., Leen A., Chai S.J., Petrochelli V., Chintagumpala M., Hurwitz M.Y., Chévez-Barrios P., Hurwitz R.L. (2010). Absence of Systemic Immune Response to Adenovectors After Intraocular Administration to Children with Retinoblastoma. Mol. Ther..

[131] Song X., Zhou Y., Jia R., Xu X., Wang H., Hu J., Ge S., Fan X. (2010). Inhibition of Retinoblastoma In Vitro and In Vivo with Conditionally Replicating Oncolytic Adenovirus H101. Investig. Ophthalmol. Vis. Sci..

[132] Song X., Wang H., Jia R., Cun B., Zhao X., Zhou Y., Xu X., Qian G., Ge S., Fan X. (2012). Combined Treatment with an Oncolytic Adenovirus and Antitumor Activity of Vincristine against Retinoblastoma Cells. Int. J. Mol. Sci..

[133] Durham N.M., Mulgrew K., McGlinchey K., Monks N.R., Ji H., Herbst R., Suzich J., Hammond S.A., Kelly E.J. (2017). Oncolytic VSV Primes Differential Responses to Immuno-oncology Therapy. Mol. Ther..

[134] Reddy P.S., Burroughs K.D., Hales L.M., Ganesh S., Jones B.H., Idamakanti N., Hay C., Li S.S., Skele K.L., Vasko A.-J. (2007). Seneca Valley Virus, a Systemically Deliverable Oncolytic Picornavirus, and the Treatment of Neuroendocrine Cancers. J. Natl. Cancer Inst..

[135] Wadhwa L., Hurwitz M.Y., Chévez-Barrios P., Hurwitz R.L. (2007). Treatment of Invasive Retinoblastoma in a Murine Model Using an Oncolytic Picornavirus. Cancer Res..

[136] Burke M.J., Ahern C., Weigel B.J., Poirier J.T., Rudin C.M., Chen Y., Cripe T.P., Bernhardt M.B., Blaney S.M. (2015). Phase I trial of Seneca Valley Virus (NTX-010) in children with relapsed/refractory solid tumors: A report of the Children’s Oncology Group. Pediatr. Blood Cancer.

[137] Aura Biosciences (2022). A Phase 2 Open-Label, Ascending Single and Repeat Dose Escalation Trial of Belzupacap Sarotalocan (AU-011) via Suprachoroidal Administration in Subjects with Primary Indeterminate Lesions and Small Choroidal Melanoma. https://aurabiosciences.com/wp-content/uploads/2023/02/Macula-Society-2023-SC-Administration-2022_02_16_FINAL-as-presented.pdf.

[138] Savinainen A., Grossniklaus H., Kang S., Rasmussen C., Bentley E., Krakova Y., Struble C.B., Rich C. (2020). Ocular distribution and efficacy after suprachoroidal injection of AU-011 for treatment of ocular melanoma. Investig. Ophthalmol. Vis. Sci..

[139] Savinainen A., Grossniklaus H.E., King S., Wicks J., Rich C.C. (2021). Ocular distribution and exposure of AU-011 after suprachoroidal or intravitreal administration in an orthotopic rabbit model of human uveal melanoma. Investig. Ophthalmol. Vis. Sci..

[140] Mruthyunjaya P., Schefler A.C., Kim I.K., Bergstrom C., Demirci H., Tsai T., Bhavsar A.R., Capone A., Marr B., McCannel T.A. (2020). A Phase 1b/2 Open-label Clinical Trial to Evaluate the Safety and Efficacy of AU-011 for the Treatment of Choroidal Melanoma. Investig. Ophthalmol. Vis. Sci..

[141] McCannel T.A., Bhavsar A., Capone A., Demirici H., Kim I.K., Marr B., Rich C., Schefler A.C., Shields C.L. (2019). Two year results of a phase 1b/2 open-label clinical trial of AU-011 for the treatment of small to medium choroidal melanoma. Investig. Ophthalmol. Vis. Sci..

[142] Demirci H., Narvekar A., Murray C., Rich C. (2022). 842P A phase II trial of AU-011, an investigational, virus-like drug conjugate (VDC) for the treatment of primary indeterminate lesions and small choroidal melanoma (IL/CM) using suprachoroidal administration. Ann. Oncol..

[143] Wojtukiewicz M.Z., Rek M.M., Karpowicz K., Górska M., Polityńska B., Wojtukiewicz A.M., Moniuszko M., Radziwon P., Tucker S.C., Honn K.V. (2021). Inhibitors of immune checkpoints—PD-1, PD-L1, CTLA-4—New opportunities for cancer patients and a new challenge for internists and general practitioners. Cancer Metastasis Rev..

[144] Bai H., Bosch J.J., Heindl L.M. (2023). Current management of uveal melanoma: A review. Clin. Exper Ophthalmol..

[145] Strobel S.B., Machiraju D., Hassel J.C. (2022). TCR-Directed Therapy in the Treatment of Metastatic Uveal Melanoma. Cancers.

[146] Ribas A., Wolchok J.D. (2018). Cancer immunotherapy using checkpoint blockade. Science.

[147] Hazarika M., Chuk M.K., Theoret M.R., Mushti S., He K., Weis S.L., Putman A.H., Helms W.S., Cao X., Li H. (2017). U.S. FDA Approval Summary: Nivolumab for Treatment of Unresectable or Metastatic Melanoma Following Progression on Ipilimumab. Clin. Cancer Res..

[148] Garon E.B., Rizvi N.A., Hui R., Leighl N., Balmanoukian A.S., Eder J.P., Patnaik A., Aggarwal C., Gubens M., Horn L. (2015). Pembrolizumab for the Treatment of Non–Small-Cell Lung Cancer. N. Engl. J. Med..

[149] Jain S., Clark J.I. (2015). Ipilimumab for the Treatment of Melanoma. Melanoma Manag..

[150] Ahmadzadeh M., Johnson L.A., Heemskerk B., Wunderlich J.R., Dudley M.E., White D.E., Rosenberg S.A. (2009). Tumor antigen–specific CD8 T cells infiltrating the tumor express high levels of PD-1 and are functionally impaired. Blood.

[151] Zhan M.-M., Hu X.-Q., Liu X.-X., Ruan B.-F., Xu J., Liao C. (2016). From monoclonal antibodies to small molecules: The development of inhibitors targeting the PD-1/PD-L1 pathway. Drug Discov. Today.

[152] Mansh M. (2011). Ipilimumab and cancer immunotherapy: A new hope for advanced stage melanoma. Yale J. Biol. Med..

[153] Buchbinder E.I., Desai A. (2016). CTLA-4 and PD-1 Pathways: Similarities, Differences, and Implications of Their Inhibition. Am. J. Clin. Oncol..

[154] Salvi S., Fontana V., Boccardo S., Merlo D.F., Margallo E., Laurent S., Morabito A., Rijavec E., Dal Bello M.G., Mora M. (2012). Evaluation of CTLA-4 expression and relevance as a novel prognostic factor in patients with non-small cell lung cancer. Cancer Immunol. Immunother..

[155] Huang P.-Y., Guo S.-S., Zhang Y., Lu J.-B., Chen Q.-Y., Tang L.-Q., Zhang L., Liu L.-T., Zhang L., Mai H.-Q. (2016). Tumor CTLA-4 overexpression predicts poor survival in patients with nasopharyngeal carcinoma. Oncotarget.

[156] Qureshi O.S., Zheng Y., Nakamura K., Attridge K., Manzotti C., Schmidt E.M., Baker J., Jeffery L.E., Kaur S., Briggs Z. (2011). Trans-Endocytosis of CD80 and CD86: A Molecular Basis for the Cell-Extrinsic Function of CTLA-4. Science.

[157] Kirkwood J.M., Butterfield L.H., Tarhini A.A., Zarour H., Kalinski P., Ferrone S. (2012). Immunotherapy of cancer in 2012. CA A Cancer J. Clin..

[158] Sobhani N., Tardiel-Cyril D.R., Davtyan A., Generali D., Roudi R., Li Y. (2021). CTLA-4 in Regulatory T Cells for Cancer Immunotherapy. Cancers.

[159] Ruella M., Kalos M. (2014). Adoptive immunotherapy for cancer. Immunol. Rev..

[160] Baruch E.N., Berg A.L., Besser M.J., Schachter J., Markel G. (2017). Adoptive T cell therapy: An overview of obstacles and opportunities. Cancer.

[161] Sterner R.C., Sterner R.M. (2021). CAR-T cell therapy: Current limitations and potential strategies. Blood Cancer J..

[162] Martinez M., Moon E.K. (2019). CAR T Cells for Solid Tumors: New Strategies for Finding, Infiltrating, and Surviving in the Tumor Microenvironment. Front. Immunol..

[163] Yan T., Zhu L., Chen J. (2023). Current advances and challenges in CAR T-Cell therapy for solid tumors: Tumor-associated antigens and the tumor microenvironment. Exp. Hematol. Oncol..

[164] Middleton M.R., McAlpine C., Woodcock V.K., Corrie P., Infante J.R., Steven N.M., Evans T.R.J., Anthoney A., Shoushtari A.N., Hamid O. (2020). Tebentafusp, A TCR/Anti-CD3 Bispecific Fusion Protein Targeting gp100, Potently Activated Antitumor Immune Responses in Patients with Metastatic Melanoma. Clin. Cancer Res..

[165] Garrido F. (2019). MHC/HLA Class I Loss in Cancer Cells. MHC Class-I Loss and Cancer Immune Escape.

[166] Boudousquie C., Bossi G., Hurst J.M., Rygiel K.A., Jakobsen B.K., Hassan N.J. (2017). Polyfunctional response by Imm TAC (IMC gp100) redirected CD8^+^ and CD4^+^ T cells. Immunology.

[167] Wespiser M., Neidhardt E., Negrier S. (2023). Uveal melanoma: In the era of new treatments. Cancer Treat. Rev..

[168] Olivier T., Haslam A., Tuia J., Prasad V. (2023). Eligibility for Human Leukocyte Antigen–Based Therapeutics by Race and Ethnicity. JAMA Netw. Open.

[169] Shahid K., Khalife M., Dabney R., Phan A.T. (2019). Immunotherapy and targeted therapy-the new roadmap in cancer treatment. Ann. Transl. Med..

[170] Thomas M., Armenti S.T., Ayres M.B., Demirci H. (2018). Uveal Effusion After Immune Checkpoint Inhibitor Therapy. JAMA Ophthalmol..

[171] Zhou Y.-W., Xu Q., Wang Y., Xia R.-L., Liu J.-Y., Ma X.-L. (2022). Immune checkpoint inhibitor-associated ophthalmic adverse events: Current understanding of its mechanisms, diagnosis, and management. Int. J. Ophthalmol..

[172] Ganesan B., Parameswaran S., Sharma A., Krishnakumar S. (2020). Clinical relevance of B7H3 expression in retinoblastoma. Sci. Rep..

[173] Zhao B., Li H., Xia Y., Wang Y., Wang Y., Shi Y., Xing H., Qu T., Wang Y., Ma W. (2022). Immune checkpoint of B7-H3 in cancer: From immunology to clinical immunotherapy. J. Hematol. Oncol..

[174] Kovaleva O.V., Belova T.P., Korotkova E.A., Kushlinskii D.N., Gratchev A.N., Petrikova N.A., Kudlay D.A., Kushlinskii N.E. (2021). Soluble B7-H3 in Ovarian Cancer and Its Predictive Value. Bull. Exp. Biol. Med..

[175] Li Y., Cai Q., Shen X., Chen X., Guan Z. (2021). Overexpression of B7-H3 Is Associated With Poor Prognosis in Laryngeal Cancer. Front. Oncol..

[176] Mao Y., Li W., Chen K., Xie Y., Liu Q., Yao M., Duan W., Zhou X., Liang R., Tao M. (2015). B7-H1 and B7-H3 are independent predictors of poor prognosis in patients with non-small cell lung cancer. Oncotarget.

[177] Altan M., Pelekanou V., Schalper K.A., Toki M., Gaule P., Syrigos K., Herbst R.S., Rimm D.L. (2017). B7-H3 Expression in NSCLC and Its Association with B7-H4, PD-L1 and Tumor-Infiltrating Lymphocytes. Clin. Cancer Res..

[178] Lu Z., Zhao Z.-X., Cheng P., Huang F., Guan X., Zhang M.-G., Chen H.-P., Liu Z., Jiang Z., Zheng Z.-X. (2020). B7-H3 immune checkpoint expression is a poor prognostic factor in colorectal carcinoma. Mod. Pathol..

[179] Wang L., Li S., Mei J., Ye L. (2022). Immunotherapies of retinoblastoma: Effective methods for preserving vision in the future. Front. Oncol..

[180] Philippova J., Shevchenko J., Sennikov S. (2024). GD2-targeting therapy: A comparative analysis of approaches and promising directions. Front. Immunol..

[181] Schengrund C.-L. (2023). The Ying and Yang of Ganglioside Function in Cancer. Cancers.

[182] Nazha B., Inal C., Owonikoko T.K. (2020). Disialoganglioside GD2 Expression in Solid Tumors and Role as a Target for Cancer Therapy. Front. Oncol..

[183] Machy P., Mortier E., Birklé S. (2023). Biology of GD2 ganglioside: Implications for cancer immunotherapy. Front. Pharmacol..

[184] Theruvath J., Menard M., Smith B.A.H., Linde M.H., Coles G.L., Dalton G.N., Wu W., Kiru L., Delaidelli A., Sotillo E. (2022). Anti-GD2 synergizes with CD47 blockade to mediate tumor eradication. Nat. Med..

[185] Mokbel K. (2024). GD2 in Breast Cancer: A Potential Biomarker and Therapeutic Target. Cancer Genom. Proteom..

[186] Higashi C., Saito K., Kozuka Y., Yuasa H., Nakamura K., Ishitobi M., Ishihara M., Mizuno T., Tawara I., Fujiwara H. (2023). Ganglioside GD2 Expression Is Associated With Unfavorable Prognosis in Early Triple-negative Breast Cancer. Anticancer Res..

[187] Portoukalian J., David M.J., Gain P., Richard M. (1993). Shedding of GD2 ganglioside in patients with retinoblastoma. Int. J. Cancer.

[188] Laurent V.E., Sampor C., Solernou V., Rossi J., Gabri M., Eandi-Eberle S., de Davila M.T.G., Alonso D.F., Chantada G.L. (2013). Detection of minimally disseminated disease in the cerebrospinal fluid of children with high-risk retinoblastoma by reverse transcriptase-polymerase chain reaction for GD2 synthase mRNA. Eur. J. Cancer.

[189] Wei A.Z., Maniar A.B., Carvajal R.D. (2022). New targeted and epigenetic therapeutic strategies for the treatment of uveal melanoma. Cancer Gene Ther..

[190] Carvajal R.D., Butler M.O., Shoushtari A.N., Hassel J.C., Ikeguchi A., Hernandez-Aya L., Nathan P., Hamid O., Piulats J.M., Rioth M. (2022). Clinical and molecular response to tebentafusp in previously treated patients with metastatic uveal melanoma: A phase 2 trial. Nat. Med..

[191] Ny L., Jespersen H., Karlsson J., Alsén S., Filges S., All-Eriksson C., Andersson B., Carneiro A., Helgadottir H., Levin M. (2021). The PEMDAC phase 2 study of pembrolizumab and entinostat in patients with metastatic uveal melanoma. Nat. Commun..

[192] Kraehenbuehl L., Holland A., Armstrong E., O’Shea S., Mangarin L., Chekalil S., Johnston A., Bomalaski J.S., Erinjeri J.P., Barker C.A. (2022). Pilot Trial of Arginine Deprivation Plus Nivolumab and Ipilimumab in Patients with Metastatic Uveal Melanoma. Cancers.

[193] Piulats J.M., Espinosa E., De La Cruz Merino L., Varela M., Alonso Carrión L., Martín-Algarra S., López Castro R., Curiel T., Rodríguez-Abreu D., Redrado M. (2021). Nivolumab Plus Ipilimumab for Treatment-Naïve Metastatic Uveal Melanoma: An Open-Label, Multicenter, Phase II Trial by the Spanish Multidisciplinary Melanoma Group (GEM-1402). J. Clin. Oncol..

[194] Joshua A.M., Monzon J.G., Mihalcioiu C., Hogg D., Smylie M., Cheng T. (2015). A phase 2 study of tremelimumab in patients with advanced uveal melanoma. Melanoma Res..

[195] Amaria R.N., Postow M., Burton E.M., Tetzlaff M.T., Ross M.I., Torres-Cabala C., Glitza I.C., Duan F., Milton D.R., Busam K. (2022). Neoadjuvant relatlimab and nivolumab in resectable melanoma. Nature.

[196] Pelster M.S., Gruschkus S.K., Bassett R., Gombos D.S., Shephard M., Posada L., Glover M.S., Simien R., Diab A., Hwu P. (2021). Nivolumab and Ipilimumab in Metastatic Uveal Melanoma: Results From a Single-Arm Phase II Study. J. Clin. Oncol..

[197] Nathan P., Hassel J.C., Rutkowski P., Baurain J.-F., Butler M.O., Schlaak M., Sullivan R.J., Ochsenreither S., Dummer R., Kirkwood J.M. (2021). Overall Survival Benefit with Tebentafusp in Metastatic Uveal Melanoma. N. Engl. J. Med..

[198] Maio M., Danielli R., Chiarion-Sileni V., Pigozzo J., Parmiani G., Ridolfi R., De Rosa F., Del Vecchio M., Di Guardo L., Queirolo P. (2013). Efficacy and safety of ipilimumab in patients with pre-treated, uveal melanoma. Ann. Oncol..

[199] Sacco J.J., Harrington K.J., Olsson-Brown A., Chan T.Y., Nenclares P., Leslie I., Bommareddy P., Kalbasi A., Xie B., Mishal M. (2024). Safety, efficacy, and biomarker results from an open-label, multicenter, phase 1 study of RP2 alone or combined with nivolumab in a cohort of patients with uveal melanoma. J. Clin. Oncol..

[200] Lu R.-M., Hwang Y.-C., Liu I.-J., Lee C.-C., Tsai H.-Z., Li H.-J., Wu H.-C. (2020). Development of therapeutic antibodies for the treatment of diseases. J. Biomed. Sci..

[201] Larkin J., Chiarion-Sileni V., Gonzalez R., Grob J.J., Cowey C.L., Lao C.D., Schadendorf D., Dummer R., Smylie M., Rutkowski P. (2015). Combined Nivolumab and Ipilimumab or Monotherapy in Untreated Melanoma. N. Engl. J. Med..

[202] Harper J., Adams K.J., Bossi G., Wright D.E., Stacey A.R., Bedke N., Martinez-Hague R., Blat D., Humbert L., Buchanan H. (2018). An approved in vitro approach to preclinical safety and efficacy evaluation of engineered T cell receptor anti-CD3 bispecific (ImmTAC) molecules. PLoS ONE.

[203] Cao L., Chen S., Sun R., Ashby C.R.J., Wei L., Huang Z., Chen Z.-S. (2023). Darovasertib, a novel treatment for metastatic uveal melanoma. Front. Pharmacol..

[204] Joshua A.M., O’day R., Glasson W., Sia D., McGrath L., Ameratunga M., Cosman R., Cherepanoff S., O’Quigley M., Beaupre D.M. (2024). A phase 2 safety and efficacy study of neoadjuvant/adjuvant darovasertib for localized ocular melanoma. J. Clin. Oncol..

[205] Gezgin G., Visser M., Ruano D., Santegoets S.J., De Miranda N.F.C.C., Van Der Velden P.A., Luyten G.P.M., Van Der Burg S.H., Verdegaal E.M., Jager M.J. (2022). Tumor-Infiltrating T Cells Can Be Expanded Successfully from Primary Uveal Melanoma after Separation from Their Tumor Environment. Ophthalmol. Sci..

[206] Eichholz T., Heubach F., Arendt A.-M., Seitz C., Brecht I.B., Ebinger M., Flaadt T., Süsskind D., Richter L., Hülsenbeck I. (2024). Targeted therapies in retinoblastoma: GD2-directed immunotherapy following autologous stem cell transplantation and evaluation of alternative target B7-H3. Cancer Immunol. Immunother..

[207] Sujjitjoon J., Sayour E., Tsao S.-T., Uiprasertkul M., Sanpakit K., Buaboonnam J., Yenchitsomanus P., Atchaneeyasakul L., Chang L.-J. (2021). GD2-specific chimeric antigen receptor-modified T cells targeting retinoblastoma—Assessing tumor and T cell interaction. Transl. Oncol..

[208] Bottino C., Vitale C., Dondero A., Castriconi R. (2023). B7-H3 in Pediatric Tumors: Far beyond Neuroblastoma. Cancers.

[209] Flem-Karlsen K., Fodstad Ø., Tan M., Nunes-Xavier C.E. (2018). B7-H3 in Cancer—Beyond Immune Regulation. Trends Cancer.

[210] Ravanpay A.C., Gust J., Johnson A.J., Rolczynski L.S., Cecchini M., Chang C.A., Hoglund V.J., Mukherjee R., Vitanza N.A., Orentas R.J. (2019). EGFR806-CAR T cells selectively target a tumor-restricted EGFR epitope in glioblastoma. Oncotarget.

[211] Shalhout S.Z., Miller D.M., Emerick K.S., Kaufman H.L. (2023). Therapy with oncolytic viruses: Progress and challenges. Nat. Rev. Clin. Oncol..

[212] Robilotti E.V., Kumar A., Glickman M.S., Kamboj M. (2019). Viral oncolytic immunotherapy in the war on cancer: Infection control considerations. Infect. Control Hosp. Epidemiol..

[213] Harrington K.J., Michielin O., Malvehy J., Pezzani Grüter I., Grove L., Frauchiger A.L., Dummer R. (2017). A practical guide to the handling and administration of talimogene laherparepvec in Europe. Onco Targets Ther..

[214] Gutzmer R., Harrington K.J., Hoeller C., Lebbé C., Malvehy J., Öhrling K., Downey G., Dummer R. (2018). Practical clinical guide on the use of talimogene laherparepvec monotherapy in patients with unresectable melanoma in Europe. Eur. J. Dermatol..

[215] McBride A., Valgus J., Parsad S., Sommermann E.M., Nunan R. (2018). Pharmacy operationalization of the intralesional oncolytic immunotherapy talimogene laherparepvec. Hosp. Pharm..

[216] Evans R.K., Nawrocki D.K., Isopi L.A., Williams D.M., Casimiro D.R., Chin S., Chen M., Zhu D.-M., Shiver J.W., Volkin D.B. (2004). Development of stable liquid formulations for adenovirus-based vaccines. J. Pharm. Sci..

[217] Schirrmacher V. (2020). Cancer Vaccines and Oncolytic Viruses Exert Profoundly Lower Side Effects in Cancer Patients than Other Systemic Therapies: A Comparative Analysis. Biomedicines.

[218] Mielgo A., Schmid M.C. (2020). Liver Tropism in Cancer: The Hepatic Metastatic Niche. Cold Spring Harb. Perspect. Med..

[219] Zheng M., Tian Z. (2019). Liver-Mediated Adaptive Immune Tolerance. Front. Immunol..

